# Regulation of neuropathic pain by microglial Orai1 channels

**DOI:** 10.1126/sciadv.ade7002

**Published:** 2023-01-27

**Authors:** Shogo Tsujikawa, Kaitlyn E. DeMeulenaere, Maria V. Centeno, Shahrzad Ghazisaeidi, Megan E. Martin, Martinna R. Tapies, Mohammad M. Maneshi, Megumi Yamashita, Kenneth A. Stauderman, Apkar V. Apkarian, Michael W. Salter, Murali Prakriya

**Affiliations:** ^1^ Department of Pharmacology, Northwestern University Feinberg School of Medicine, Chicago, IL 60611, USA.; ^2^ Department of Physiology, Northwestern University Feinberg School of Medicine, Chicago, IL 60611, USA.; ^3^ Hospital for Sick Children Research Institute, Toronto, Canada.; ^4^ CalciMedica Inc., 505 Coast Blvd. South, Suite 202, La Jolla, CA 92037, USA.

## Abstract

Microglia are important mediators of neuroinflammation, which underlies neuropathic pain. However, the molecular checkpoints controlling microglial reactivity are not well-understood. Here, we investigated the role of Orai1 channels for microglia-mediated neuroinflammation following nerve injury and find that deletion of Orai1 in microglia attenuates Ca^2+^ signaling and the production of inflammatory cytokines by proalgesic agonists. Conditional deletion of Orai1 attenuated microglial proliferation in the dorsal horn, spinal cytokine levels, and potentiation of excitatory neurotransmission following peripheral nerve injury. These cellular effects were accompanied by mitigation of pain hyperalgesia in microglial Orai1 knockout mice. A small-molecule Orai1 inhibitor, CM4620, similarly mitigated allodynia in male mice. Unexpectedly, these protective effects were not seen in female mice, revealing sexual dimorphism in Orai1 regulation of microglial reactivity and hyperalgesia. Together, these findings indicate that Orai1 channels are key regulators of the sexually dimorphic role of microglia for the neuroinflammation that underlies neuropathic pain.

## Teaser

Orai1 calcium channels in microglia ﻿control neuropathic pain in asex-specific manner.

## INTRODUCTION

Microglia are the tissue-resident macrophage cells of the nervous system comprising about 5 to 10% of the cells in the central nervous system (CNS) (*1*). Widely considered to be the sentinels of the brain, microglia in the healthy brain survey their environment for changes in synaptic activity, tissue damage, and infections via their highly ramified processes. Following brain damage or infection, however, microglia are activated rapidly via a process termed microgliosis to mediate several additional major roles. First, they promote neuronal well-being by removing debris and dying cells via phagocytosis and efferocytosis (*1*, *2*). Second, they defend against infections and tissue damage by secreting proinflammatory and proresolving mediators (*3*). Although these dueling functions are well balanced in the healthy brain, under pathological conditions, the failure of checkpoints in inflammatory signaling cascades can transform microglia into a disease-associated form, causing persistent, damaging neuroinflammation (*2*, *4*). In this study, we examine a potential role for store-operated Orai1 channels in regulating inflammatory functions of activated microglia in the context of neuropathic pain.

Microglial reactivity following nerve injury is driven by a variety of membrane receptors, but purinergic receptors including the P2X and P2Y receptor families (*4*–*7*) have received especially strong scrutiny in microglial activation after nerve injury. Prevailing views indicate that stimulation of these receptors by adenosine triphosphate (ATP) turns on the p38 mitogen-activated protein (MAP) kinase and nuclear factor κB (NF-κB) pathways in microglia, triggering synthesis and release of a variety of proinflammatory agents including interleukin-6 (IL-6), tumor necrosis factor–α (TNFα), and IL-1β, which potentiate excitatory synaptic transmission in the dorsal spinal horn and remodel spinal circuits involved in neuropathic pain (*3*, *4*, *8*). In line with this evidence, inhibiting proinflammatory cytokines by broadly targeting microglial activation with tetracycline antibiotics such as minocycline, or destroying microglia with immunotoxins reduce allodynia and other end points of neuropathic pain (*9*–*14*). Puzzlingly, however, microglial contributions to neuropathic pain are highly sex-dependent. Attenuation of hypersensitivity and allodynia by broad-spectrum microglial inhibitors and microglial toxins is reported to occur in male but not female mice (*12*, *15*–*18*). Whether this sex difference is due to sex-dependent variations in the spinal neuronal responses to nerve injury, spinal cytokine levels, or cell-autonomous differences in microglia is widely debated and not well understood (*17*, *18*). More generally, despite the explosion of information about neuropathic pain in recent years, no breakthrough treatments have emerged and even the mechanisms of the pathogenesis itself are not fully established (*19*). The slow progress probably reflects a combination of factors including inadequate genetic tools for isolating specific microglial signaling pathways, and the multifactorial nature of neuropathic pain with interactions between multiple types of cells, cytokines, intrinsic abnormalities, and environmental factors.

As electrically nonexcitable cells, microglial excitability is primarily regulated by cellular Ca^2+^ elevations evoked by cell surface receptors (*20*, *21*). These Ca^2+^ signals stimulate numerous microglial functions including phagocytosis, calcineurin/nuclear factor of activated T (NFAT) signaling, and Toll-like receptor (TLR4)–mediated NF-κB activation (*20*, *22*, *23*). Ca^2+^ regulation of these processes is particularly well suited for its potential to be stimulated rapidly and modulated by other signaling pathways. However, our understanding of the pathways mediating microglial Ca^2+^ signaling and their contributions to microglial reactivity and neuroinflammation remains poorly understood. There is evidence for Ca^2+^ release from intracellular stores (*20*). However, because release of Ca^2+^ from intracellular stores is typically coupled to activation of store-operated Ca^2+^ entry (SOCE) (*24*) and previous studies were not designed to discriminate between SOCE and store release, the specific physiological contributions of SOCE for microglial reactivity and neuroinflammation have not been critically evaluated in past work.

In most animal cells, SOCE serves as a central mechanism for mobilizing [Ca^2+^]_i_ elevations and is activated by the engagement of heterotrimeric G protein, or tyrosine kinase receptors (*24*). The ensuing depletion of endoplasmic reticulum (ER) stores stimulates the ER Ca^2+^ sensors, the stromal interaction molecules (STIM) 1/2, which, in turn, activate Ca^2+^ release–activated Ca^2+^ (CRAC) channels formed by the Orai proteins (*24*). Orai1, the best studied member of this family, is exquisitely Ca^2+^ selective, making it ideally suited for generating oscillatory and long-lasting Ca^2+^ signals needed for transcriptional and enzymatic cascades (*24*, *25*). In the nervous system, Orai1 is implicated in a growing list of functions including gliotransmitter release, synaptic plasticity, phagocytosis, chemotactic migration, and activation of NF-κB and MAP kinase (*26*–*29*). Recently, a whole-animal Orai1 knockout (KO) was shown to be resistant to carrageenan- and formalin-induced pain, which was attributed to changes in neuronal excitability, especially in the dorsal horn neurons of the spinal cord (*30*). However, the contribution of microglial Orai1 channels for neuroinflammation that underlies neuropathic pain is unknown.

These findings led us to consider several questions. Can manipulation of microglial activation by blocking SOCE affect microglial inflammation following nerve injury? How does this affect excitatory synaptic transmission in the spinal cord? And what are the implications for neuropathic pain? In this study, we addressed these questions using a microglial-specific Orai1 KO mouse and biochemical, electrophysiological, and behavioral assays. We find that Orai1 channels in microglia are critical for the synthesis and release of inflammatory mediators and play a central role in the potentiation of synaptic transmission in the spinal cord following nerve injury and the mechanical pain hypersensitivity that is the hallmark of neuropathic pain.

## RESULTS

### Orai1 is essential for SOCE in microglia

To address the role of microglial Orai1 channels in regulating microglial Ca^2+^ signaling, neuroinflammation, and neuropathic pain following peripheral nerve injury, we generated a microglial-specific Orai1 KO mouse line by crossing *Orai1^fl/fl^* mice with the inducible Cre line, *Cx3CR1-Cre/ERT2*, for selective deletion of Orai1 in microglia in the CNS (*31*). Homozygous *Orai1^fl/fl Cx3CR1-Cre/ERT2^* mice arising from this cross were born at Mendelian ratios and did not differ from wild-type (WT) mice in terms of weight, litter size, or gross mobility. To knock out Orai1 expression, we used two approaches: (i) For the in vitro mechanistic studies, we isolated primary spinal cord microglia from *Orai1^fl/fl Cx3CR1-Cre/ERT2^* mice or WT controls from postnatal day 0 (P0) to P2 stage, cultured them for 10 days, and added tamoxifen or vehicle to the culture medium for Orai1 deletion (see Materials and Methods). Reverse transcription quantitative polymerase chain reaction (RT-qPCR) analysis indicated that after tamoxifen treatment, microglia from *Orai1^fl/fl Cx3CR1-Cre/ERT2^* mice showed marked loss of Orai1 mRNA (fig. S1, A and B), indicating that Cre recombination is highly successful in deleting Orai1 in the cultured microglia. (ii) For the in vivo studies, we intraperitoneally injected young adult (~5 weeks of age) mice with tamoxifen or corn oil (as controls) and used the mice for experiments 14 days (or for the experiment in fig. S5, 34 days) later. RT-qPCR analysis of freshly isolated microglia isolated 21 days after tamoxifen administration from adult mice (fig. S1C) showed marked loss of Orai1 mRNA with no change in the expression of Orai2 and Orai3 isoforms or in the expression of STIM1/2, indicating that this technique provides a highly effective approach for selectively deleting Orai1 in brain microglia (fig. S1D). These microglial Orai1-specific conditional KO cells and tamoxifen-injected mice will henceforth be referred to as Orai1 cKO cells/mice. *Orai1^fl/fl^* mice and *Orai1^fl/fl Cx3CR1-Cre/ERT2^* mice exposed to the vehicle (corn oil) were used as controls.

**Fig. 1.**
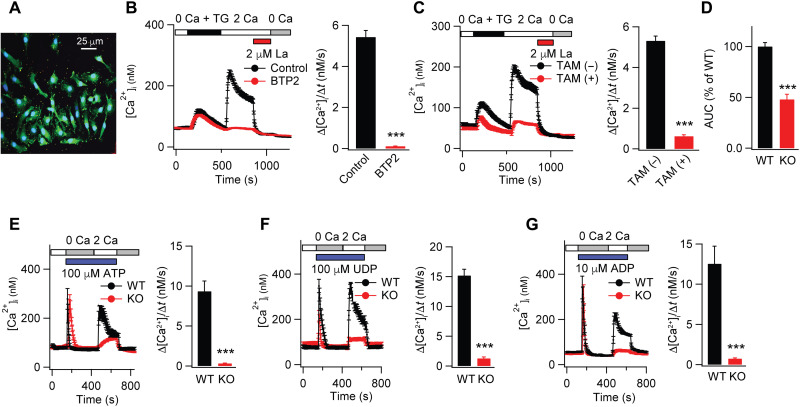
Deletion of Orai1 abrogates SOCE in spinal cord microglia. (**A**) Primary cells cultured from spinal cord were immunolabeled with the microglial/macrophage marker, IBA1. Blue, 4′,6-diamidino-2-phenylindole (DAPI); green, IBA1. (**B**) SOCE was induced by depleting ER Ca^2+^ stores with TG (1 μM) in Ca^2+^-free solution followed by readdition of extracellular Ca^2+^ (2 mM). SOCE is blocked by the CRAC channel inhibitors, La^3+^ and BTP2 (1 μM). The bar graph on the right summarizes the rate of Ca^2+^ influx measured by the initial slope of [Ca^2+^]_i_ rise over 18 s after Ca^2+^ readdition. *n* = 43 cells, control; *n* = 56 cells, BTP2 (mean ± SEM). (**C**) SOCE is abrogated by the deletion of Orai1. Microglia from *Orai1^fl/fl Cx3CR1-Cre/ERT2^* mice were cultured either in the absence or presence of tamoxifen. Bar graphs summarize the rate of SOCE following addback of extracellular Ca^2+^. *n* = 56 cells, no tamoxifen; *n* = 45 cells, with tamoxifen. (**D**) Intracellular Ca^2+^ store release is diminished in Orai1 KO microglia. Store release was assessed by quantifying the area under the curve (AUC) during TG application in the Ca^2+^-free solution until the start time of extracellular Ca^2+^ readdition (378 s) and normalized to WT levels. *n* = 56 cells, no tamoxifen; *n* = 45 cells, tamoxifen. (**E**) Stimulating microglia with ATP (100 μM) in Ca^2+^-free solution evokes store release, followed by SOCE when extracellular Ca^2+^ is restored. SOCE is strongly reduced in the Orai1 cKO cells. *n* = 35 cells, WT + ATP; *n* = 50 cells, Orai1 cKO + ATP. (**F** and **G**) Likewise, SOCE evoked by the selective P2Y_6_ receptor agonist, UDP (100 μM), and the P2Y_12_ receptor agonist, ADP (10 μM) is abrogated in Orai cKO microglia. UDP: *n* = 35 (WT) and *n* = 39 cells (Orai1 KO). ADP: *n* = 48 (WT) and *n* = 58 (Orai1 KO). In each case, the right graphs summarize the rate of Ca^2+^ influx following readdition of extracellular Ca^2+^ from three to five independent experiments. ***P < 0.001 by Mann-Whitney rank sum test.

Staining with the microglial marker ionized calcium-binding adapter molecule 1 (IBA1) revealed that primary cells isolated from the spinal cord using our isolation procedures were >90% positive for IBA1 (Fig. 1A), confirming the microglial identity of the cultured cells. To study the role of Orai1 for mediating SOCE in microglia, we loaded cells with the ratiometric dye, Fura-2/AM, and monitored cellular [Ca^2+^]_i_ elevations using a widely used Ca^2+^-addback protocol for assessing SOCE (*28*, *32*). In this protocol, we depleted ER Ca^2+^ stores with the sarcoendoplasmic reticulum Ca^2+^-ATPase (SERCA) pump inhibitor, thapsigargin (TG), administered in a Ca^2+^-free medium, and measured SOCE by examining the rate and amplitude of Ca^2+^ influx seen upon restoring extracellular Ca^2+^ (2 mM) to the bath medium (Fig. 1B). WT (*Orai1^fl/fl^*) or *Orai1^fl/fl Cx3CR1-Cre/ERT2^* microglia not exposed to tamoxifen showed robust SOCE with pharmacological properties, consistent with those of Orai1, including blockade by low doses of La^3+^ and Bis(trifluoromethyl)pyrazole 2 (BTP2) (Fig. 1, B and C) and by the recently developed Orai1 inhibitor, CM4620, which is currently in clinical trials for treating Orai1-mediated inflammatory syndromes (*33*, *34*) (fig. S2A). SOCE was similar in magnitude in the *Orai1^fl/fl Cx3CR1-Cre/ERT2^* microglia not treated with tamoxifen compared to that seen in WT (*Orai1^fl/fl^* mice) (Fig. 1, B and C), indicating that expression of Cre recombinase alone in the absence of tamoxifen has no effects on SOCE. By contrast, *Orai1^fl/fl Cx3CR1-Cre/ERT2^* microglia exposed to tamoxifen (Orai1 cKOs) showed marked loss of SOCE (Fig. 1C), with the amplitude and the rate of Ca^2+^ entry following Ca^2+^ readdition decreasing >90% (Fig. 1C). Moreover, analysis of SOCE in cells cultured separately from individual male and female pups revealed that microglia from both sexes exhibited comparable levels of SOCE (fig. S2, B and C). Deletion of Orai1 resulted in loss of SOCE in both male and female microglia (fig. S2, B and C), indicating that, in vitro, there is no sex difference in the contribution of Orai1 for mediating SOCE in microglia. Microglia lacking Orai1 also displayed reduced release of Ca^2+^ from ER Ca^2+^ stores following store depletion by TG (Fig. 1D), indicating that loss of SOCE affects store refilling as expected for a Ca^2+^ pathway responsible for recharging and replenishing stores. Together, these results indicate that Orai1 is essential for mediating SOCE in spinal cord microglia, a role congruent with its well-established role in many nonexcitable cells (*24*, *25*).

### Deletion of Orai1 impairs ATP-mediated Ca^2+^ signals in microglia

Previous studies have shown that the nucleotide neurotransmitter, ATP, which also functions as a danger-associated molecular pattern, is a potent activator of microglia inducing a variety of microglial responses including altered expression of inflammation-related genes, process retraction, migration, and increased phagocytic ability (*2*, *4*). ATP released from sensory afferents and other dorsal spinal neurons following nerve injury is thought to be a key player in the activation of microglia and induction of neuropathic pain (*5*, *35*). We have recently shown that extracellular ATP is a potent activator of Orai1-mediated SOCE in astrocytes and airway epithelial cells (*28*, *36*), raising the possibility that Orai1 channels may similarly mediate ATP-induced Ca^2+^ responses in microglia. To directly address this question, we examined Ca^2+^ signals evoked by agonists of ATP receptors. We found that administration ATP in Ca^2+^-free medium evoked robust, transient rises in [Ca^2+^]_i_ in WT microglia reflecting release of Ca^2+^ from intracellular stores (Fig. 1E). Readdition of extracellular Ca^2+^ evoked a robust secondary rise in [Ca^2+^]. The kinetic signature of this ATP-induced Ca^2+^ response is consistent with G_q_/phospholipase C-inositol 1,4,5-triphosphate signaling and SOCE that we and others have previously described in astrocytes, airway epithelial cells (AECs), and other cell types for P2Y receptors (*28*, *36*). In agreement with this interpretation, uridine diphosphate (UDP), a uridine nucleotide agonist of the P2Y6 receptor, and 2-methylthio-ADP (2-MeSADP or ADP), a ligand for the P2Y12 receptor that are both strongly implicated microglia-mediated inflammation and neuropathic pain (*6*, *7*), also evoked Ca^2+^ elevations with amplitude and kinetics comparable to those evoked by ATP (Fig. 1, F and G). Thus, these results indicate that ATP stimulates Ca^2+^ signaling in microglia at least in part through the activation of metabotropic purinergic receptors.

To examine the role of Orai1 in these Ca^2+^ responses, we examined Orai1 cKO cells in the same protocol. Deletion of Orai1 caused near-complete loss of SOCE evoked by ATP (Fig. 1E). Likewise, deletion of Orai1 impaired SOCE evoked by UDP (Fig. 1F) and by 2-MeSADP (ADP) (Fig. 1G). Together, these results indicate that CRAC channels composed of Orai1 are essential for the SOCE that is activated by stimulation of P2Y6 and P2Y12 receptors in spinal microglia.

Another receptor that is implicated in microglial activation and chronic pain is TLR4 (*37*, *38*). Intrathecal injection of the endotoxin lipopolysaccharide (LPS), a potent activator of TLR4, causes symptoms of neuropathic pain (*37*), and increasing evidence suggests that genetic or pharmacological blockade of TLR4 mitigates neuroinflammation and hyperalgesia following nerve injury (*37*, *38*). Although Ca^2+^ mobilization downstream of TLR4 activation is not widely reported, we found that application of LPS elicited an initial Ca^2+^ rise followed by slowly occurring Ca^2+^ oscillations in WT microglia that lasted for >1 hour (fig. S2D). In Orai1 cKO microglia, these low-frequency LPS-evoked Ca^2+^ fluctuations were completely abrogated, indicating that SOCE through Orai1 contributes to the slow endotoxin-mediated oscillatory Ca^2+^ signals in microglia. Together, these results indicate that Orai1 channels are a major mechanism for mobilization of Ca^2+^ influx in microglia by a variety of receptors implicated in neuropathic pain.

### Orai1 promotes production of proinflammatory cytokines from spinal microglia

**Fig. 2.**
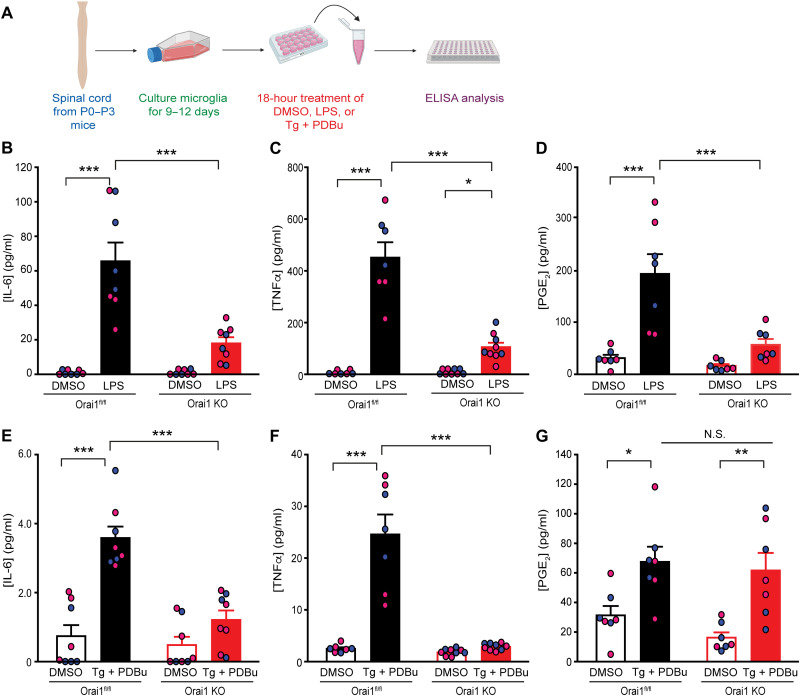
Deletion of Orai1 impairs synthesis of proinflammatory mediators from spinal microglia. (**A** to **D**) WT (*Orai1^fl/fl^*) or Orai1 cKO primary microglia cultured for 7 to 10 days and then stimulated with LPS (1 ng/ml) for 18 hours. Levels of IL-6, TNFα, and PGE_2_ were measured in the cell culture supernatant by enzyme-linked immunosorbent assay (ELISA). Cells obtained from male and female pups were cultured separately. Because of the low yield of microglia from spinal tissue, cells from two pups of the same sex were combined before the experiment. Therefore, each point represents the measurement from two animals (one flask). *n* = 7 to 9 measurements from 14 to 18 mice. ***P < 0.001 and *P < 0.05 by two-way analysis of variance (ANOVA) followed by Tukey test for comparison between multiple groups. (**E** to **G**) Cytokine measurements in microglia stimulated with TG (0.25 μM) and PDBu (25 nM). Cytokine levels were measured in the supernatant 18 hours following cell stimulation. *n* = 7 to 9 measurements from 14 to 18 mice. ***P < 0.001, **P < 0.01, and *P < 0.05 by two-way ANOVA followed by Tukey test for comparison between multiple groups. DMSO, dimethyl sulfoxide; N.S., not significant.

Microglia are known for their prolific production and release of proinflammatory cytokines, which are thought to drive neuroinflammation in disease states (*1*, *2*). Given strong links between Orai1-mediated Ca^2+^ influx and Ca^2+^-dependent gene expression of cytokines (*39*), we next sought to determine whether Orai1 has a role in mediating a similar role in microglia. We addressed this question by activating microglia with LPS, a widely used stimulus for inducing microgliosis and microglia-mediated cytokine production, or by exposing cells to TG to directly deplete Ca^2+^ stores and phorbol 12,13-dibutyrate (PDBu) to activate protein kinase C. Stimulating microglia with LPS caused strong induction of several proinflammatory mediators including IL-6, TNFα, monocyte chemotactic protein 1 (MCP1), and prostaglandin E_2_ (PGE_2_), consistent with previous evidence implicating TLR4 in stimulating microglial cytokine synthesis (Fig. 2, B to D, and fig. S3) (*40*). In Orai1 KO microglia, however, LPS-mediated induction of these proinflammatory mediators was markedly reduced (Fig. 2, B to D). By contrast, IL-10, an anti-inflammatory cytokine, was unaffected by LPS and unchanged by deletion of Orai1, suggesting that Orai1 has a stronger role in stimulating inflammatory rather than anti-inflammatory cytokines (fig. S3). Similarly, deletion of Orai1 also strongly impaired IL-6 and TNFα production from primary spinal microglia stimulated with TG + PDBu to directly activate SOCE (Fig. 2, E and F). Moreover, detailed examination of cytokines in microglia cultured separately from male and female mice showed that both the degree of induction of IL-6, TNFα, MCP1, and PGE_2_ and the effects of knocking out Orai1 were comparable in microglia isolated from male and female mice (Fig. 2). Together, these in vitro results indicate that Orai1 plays a key role in the synthesis and release of proinflammatory cytokines from spinal microglia.

### Ablation of Orai1 in microglia protects male mice against neuropathic pain

**Fig. 3.**
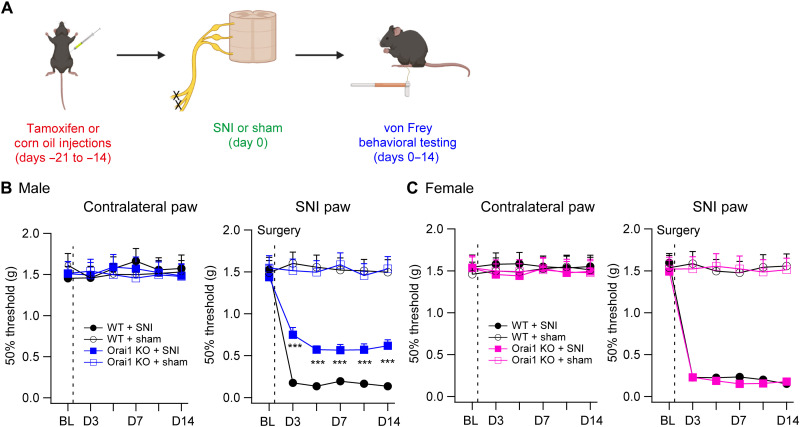
Male microglial Orai1 KO mice are partially protected in the SNI model of neuropathic pain. (**A**) A schematic of the experiment. WT (*Orai1^fl/fl^*) or Orai1 cKO (*Orai1^fl/fl Cx3CR1-Cre/ERT2^* with tamoxifen) mice (8 weeks old) were subjected to SNI or sham surgery on the left sciatic nerve, and mechanical sensitivity measured by von Frey thresholds was monitored at baseline (before SNI) and for 14 days following SNI surgery. (**B** and **C**) von Frey thresholds in male (B) or female (C) mice before and after SNI surgery in the indicated genetic groups and conditions. The dotted line indicates the day of SNI surgery. *n* values are as follows: Male WT mice: *n* = 10; male Orai1 KO mice: *n* = 10; female WT mice: *n* = 11; female Orai1 KO mice: *n* = 9. ***P < 0.001 by unpaired *t* test for each time point comparing WT and Orai1 cKO mice subjected to SNI.

Inflammatory cytokines such as TNFα and IL-6 are strongly implicated in the etiology of neuropathic pain (*10*, *14*). Thus, the finding that deletion of Orai1 reduces the production of proinflammatory cytokines from spinal microglial led us to consider the consequences for neuropathic pain. We examined this question by assessing allodynia in WT and Orai1 cKO mice following peripheral nerve injury (Fig. 3A). We used the spared nerve injury (SNI) model to induce peripheral nerve injury in young adult mice (~8 weeks), a model in which the sciatic nerve is exposed at the trifurcation of the sural, tibial, and common peroneal nerves, and the tibial and common peroneal nerves are ligated and severed, leaving the sural nerve intact (*41*, *42*). SNI is a robust model of neuropathic pain where every animal with SNI exhibits mechanical and cold allodynia for the remainder of animal life (*42*). Paw withdrawal thresholds to von Frey filament stimulation applied every 2 to 3 days were used to assess mechanical sensitivity of the hind paws before and following the SNI surgery for 14 days as previously described (*41*). All pain-related behaviors were assessed using double-blind procedures.

SNI caused paw withdrawal thresholds to markedly decrease on days 3 to 14 following the nerve injury (Fig. 3). Sham-operated mice did not show the hypersensitivity in line with previous studies, indicating that the nerve injury causes robust allodynia in this model of neuropathic pain (*42*). Orai1 cKO mice showed comparable paw withdrawal thresholds as WT mice before SNI, suggesting that microglial Orai1 channels do not directly regulate mechanical sensation. Following SNI, however, male Orai1 cKO mice showed protection against allodynia compared to sham-operated or WT (*Orai1^fl/fl^*) mice (Fig. 3B). Mitigation of allodynia in the Orai1 cKO male mice was robust, lasting >14 days following SNI surgery. *Orai1^fl/fl CX3CR1-Cre/ERT2^* mice exposed only to the vehicle (corn oil) did not show reduction in allodynia following SNI, in contrast to littermate male *Orai1^fl/fl CX3CR1-Cre/ERT2^* mice injected with tamoxifen (fig. S4), thus ruling out Cre-dependent effects in the mitigation of allodynia.

One concern in the above experiments is that because CX3CR1 is also expressed in peripheral inflammatory macrophages and monocytes, a potential role for infiltrating macrophages with loss of Orai1 cannot be formally ruled out. This possibility is moderated by the fact that monocytes and circulating macrophages are relatively short-lived and turn over rapidly within a few days (*43*, *44*), whereas resident microglia turn over extremely slowly and, moreover, are largely self-renewing (*43*, *45*). Reporter labeling studies have shown that CX3CR1-Cre mice when used 30 days or later after tamoxifen administration permit Cre-dependent recombination almost exclusively in microglia (*43*). In addition, Orai1 transcripts are undetectable in dorsal root ganglia macrophages (*46*), suggesting that these cells are unlikely to play a role in Orai1-mediated regulation of neuropathic pain. Because we observed stable and robust pain mitigation in *Orai1^fl/fl CX3CR1-Cre/ERT2^* male mice even 28 days after the last tamoxifen injection (Fig. 3B), it is unlikely that the observed phenotype is due to peripheral monocytes lacking Orai1. However, to address this concern further, we increased the duration of the waiting period before nerve injury for recombination after the final tamoxifen injection from 14 to 34 days by which time peripheral CX3CR1^+^ cell populations should be replaced by nonrecombined cells from WT progenitors. We then assessed von Frey paw withdrawal thresholds for 18 days after SNI (i.e., 52 days after the final tamoxifen injection). These experiments also showed highly significant pain mitigation in the *Orai1^fl/fl CX3CR1-Cre/ERT2^* male mice on the injured side (fig. S5). The degree of pain mitigation in Orai1 cKO mice was comparable to that seen in the cohort of mice subjected to the shorter waiting duration (Fig. 3 versus fig. S5). Thus, the most straightforward interpretation of these behavioral tests is that the loss of pain hypersensitivity in male *Orai1^fl/fl CX3CR1-Cre/ERT2^* mice is due to selective deletion of Orai1 in microglia.

In contrast to male mice, we were surprised to see that female mice behaved no differently from WT control mice (Fig. 3C). In this group, paw withdrawal thresholds after SNI in female Orai1 cKO mice were not different from those seen in *Orai1^fl/fl^* mice (Fig. 3C). Thus, protection against SNI-induced neuropathic pain in Orai1 cKO mice is highly sex specific. The finding that deletion of Orai1 in microglia produces a sex-specific phenotype similar to that previously described with microglial blockers or microglial poisoning (*12*) suggests that the sex effect is traceable to a specific signaling pathway in microglia rather than differences in downstream neural circuits in the CNS.

### Deletion of Orai1 reduces spinal cytokine levels in vivo in response to nerve injury

**Fig. 4.**
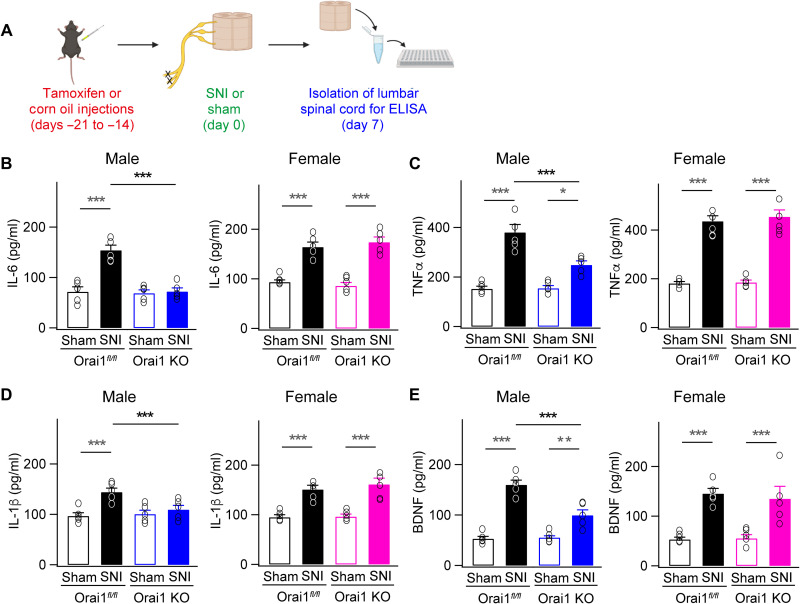
Deletion of Orai1 in microglia blocks the increases in proalgesic cytokines in the spinal cord following SNI. (**A**) A schematic of the protocol used for the experiment. WT (*Orai1^fl/fl^*) or Orai1 cKO (*Orai1^fl/fl CX3CR1-CRE/ERT2^* with tamoxifen) mice were subjected to either sham or SNI surgery. Seven days following SNI, animals were euthanized, 5 mm of the lumbar spinal cord was harvested from each mouse, and the tissue was homogenized and spun down. Levels of IL-1β, TNFα, IL-6, and the growth factor BDNF were assessed in the supernatant using ELISA. (**B** to **E**) SNI increases the levels of inflammatory cytokines and the growth factor BDNF compared to sham-operated controls. These increases are reduced in male but not female Orai1 cKO mice. (*n* = 5 mice per condition). ***P < 0.001, **P < 0.01, and *P < 0.05 by two-way ANOVA followed by Tukey test for comparison between multiple groups.

It is well established that microglia produce inflammatory cytokines that drive inflammation mediating neuropathic pain. Given that our in vitro experiments indicated that deletion of Orai1 blocked the induction of key cytokines in cultured microglia, we next considered whether the partial mitigation of mechanical allodynia in male Orai1 cKO mice is due to reduction in cytokine levels in vivo in the spinal cord following SNI. To address this question, we assessed cytokine levels in spinal cord lumbar tissue in WT and Orai1 cKO mice following peripheral nerve injury. The lumbar spinal cord (5 mm in length) at L4 to L5 at the entry of the sciatic nerve was dissected out following SNI or sham surgery from *Orai1^fl/fl^* and Orai1 cKO mice, and cytokine levels in the homogenized tissue were measured via enzyme-linked immunosorbent assay (ELISA; Fig. 4) (*14*). These measurements revealed that SNI significantly increases levels of TNFα, IL-6, IL1-β, and brain-derived neurotrophic factor (BDNF) in the spinal cords of WT mice compared to sham-operated controls (Fig. 4, B to E). Both male and female WT mice showed comparable increases in spinal cytokine levels relative to sham-operated controls following nerve injury (Fig. 4). Notably, however, the concentrations of these inflammatory cytokines were significantly reduced in male mgOrai1 mice, with IL-6, in particular, declining essentially to levels found in sham-operated controls (154 ± 10 pg/ml in SNI WT mice versus 72 ± 7 pg/ml in SNI Orai1 cKO mice; *P* < 0.001; Fig. 4B). Levels of TNFα, IL1-β, and BDNF also decreased in Orai1 cKO mice relative to WT mice following SNI (Fig. 4, C, D, and E). However, female Orai1 cKO mice subjected to SNI did not show reductions of these mediators (Fig. 4, B to E). Rather, the degree of induction of IL-6, TNFα, BDNF, and IL1- β in female Orai1 KO mice was comparable to WT mice with SNI. These results indicate that deletion of microglial Orai1 channels prevents the induction of key proinflammatory cytokines in the spinal cord that are implicated in neuropathic pain following nerve injury, but this is seen only in male mice.

### SNI-induced enhancement of glutamatergic neurotransmission in the spinal cord is partially occluded in male Orai1 cKO mice

**Fig. 5.**
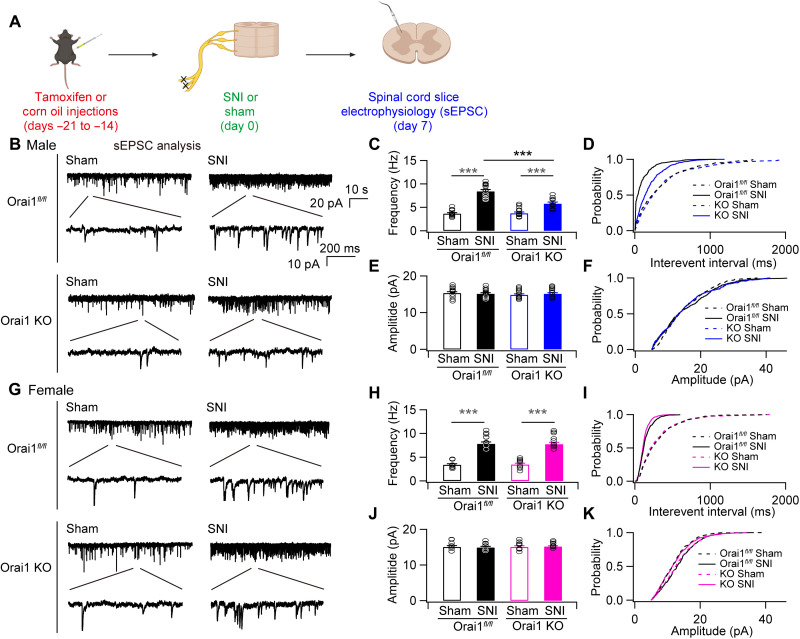
SNI-induced increase in sEPSC frequency in dorsal horn neurons is mitigated in male Orai1 cKO mice. (**A**) A schematic of the experiment. (**B**) sEPSCs recorded in the whole-cell patch-clamp configuration (−70 mV) from a lamina II neuron in the dorsal horn of the L4 spinal cord in slices from male mice. Ionotropic GABA and NMDA receptors were blocked with picrotoxin and D-APV [d-(-)-2-amino-5-phosphonopentanoic acid], respectively. The traces show examples of sEPSCs at low and high time resolutions. (**C**) The bar graph summarizes the frequency of sEPSCs in the indicated groups. Mice subjected to SNI show increase in sEPSC frequency, which is partially occluded in mgOrai1 mice. ***P < 0.001. Two-way ANOVA followed by Tukey test for comparison between multiple groups. (**D**) Cumulative probability distributions of the interevent intervals of the sEPSCs in WT and Orai1 cKO mice. The interevent interval is shifted toward lower intervals consistent with increases in the frequency of sEPSCs in mice subjected to SNI. (**E**) Summary of the sEPSC amplitudes in the indicated groups. (**F**) Cumulative distributions of the sEPSC amplitudes. (**G**) sEPSC traces in slices from female mice. (**H** and **I**) Summary of the changes in mean frequency and cumulative histogram distributions of sEPSC frequency in female mice. In female Orai1 cKO mice, the frequency of sEPSC is unaffected relative to SNI-administered WT mice. (**J** to **K**) Mean and cumulative probability distributions of the sEPSC amplitudes. *n* values are as follows: Male WT mice: *n* = 12 cells (sham) and *n* = 13 (SNI). Male Orai1 KO mice: *n* = 13 cells (sham) and *n* = 13 cells (SNI). Female WT mice: *n* = 10 cells (sham) and *n* = 10 cells (SNI). Female Orai1 KO mice: *n* = 10 cells (sham) and *n* = 10 cells (SNI). ***P < 0.001 by ANOVA followed by Tukey test for comparison of multiple groups.

Previous work has shown that peripheral nerve injury and microglial stimulation increase excitatory synaptic transmission within the lamina II neurons of the dorsal spinal cord (*47*–*49*). This maladaptive potentiation of excitatory synaptic transmission is thought to alter the set point for signal transmission in nociceptive circuits following nerve injury to mediate neuropathic pain (*3*, *4*, *8*). Thus, the finding that male Orai1 cKO mice are protected against SNI-induced allodynia (Fig. 3B) with reduced spinal cytokine levels (Fig. 4) led us to next consider whether microglial Orai1 channels regulate SNI-evoked alterations in synaptic transmission in the spinal cord. To address this question, we carried out electrophysiological analysis of spontaneous excitatory postsynaptic currents (sEPSCs) in the substantia gelatinosa (SG) neurons of the dorsal spinal cord (L4-5) following SNI (Fig. 5A). The SG region of the dorsal horn was visually identified in spinal cord slices with a water immersion objective, and sEPSCs were recorded from SG neurons in the whole-cell patch-clamp mode at −70 mV in the presence of γ-aminobutyric acid (GABA) and N-methyl-D-aspartate receptor (NMDAR) blockers as described previously (*29*, *50*).

Neuronal recordings from sham-operated WT and Orai1 cKO slices revealed no differences in the frequency or amplitude of basal sEPSC events between the two genetic groups (Fig. 5, C, E, H, and J). However, WT animals that underwent SNI exhibited notable increases in the frequency of sEPSCs, with the frequency increasing approximately twofold in SNI-operated WT mice [from 3.6 ± 0.2 Hz (*n* = 12) compared to 8.4 ± 0.38 Hz (*n* = 13; *P* < 0.001) in sham-operated controls; Fig. 5, C and H]. Cumulative histogram plots indicated that SNI significantly decreased the interevent sEPSC interval (Fig. 5, D and H), consistent with the increase in the frequency of sEPSCs. No change in the amplitude of the events, however, was detected (Fig. 5, E, F, J, and K). These changes are consistent with previous literature indicating that peripheral nerve injury increases spontaneous excitatory synaptic transmission in the lamina II neurons of the dorsal spinal cord (*47*, *49*).

In contrast to the greater than two-fold increase in sEPSC frequency seen in WT mice following SNI, male Orai1 cKO subjected to SNI showed significantly smaller potentiation of the sEPSC frequency [8.4 ± 0.38 Hz (*n* = 13) in sham-operated KOs versus 5.7 ± 0.3 (*n* = 11) in SNI animals, *P* < 0.001; Fig. 5C]. Correspondingly, Orai1 cKO mice also showed smaller changes in interevent sEPSC intervals (Fig. 5D). These results indicate that deletion of Orai1 in microglia partially prevents SNI-induced enhancement of excitatory synaptic transmission in lamina II dorsal horn neurons in male mice.

In female mice, sEPSCs in slices from WT mice subjected to SNI also showed increases in the frequency of sEPSCs [from 3.4 ± 0.2 Hz (*n* = 8) in sham-operated animals to 7.8 ± 0.4 Hz (*n* = 9; *P* < 0.001) in SNI animals; Fig. 5, H and I]. However, in contrast to male mice, female Orai1 cKO mice subjected to SNI did not show attenuation of the sEPSC frequency [3.5 ± 0.2 Hz (*n* = 9) in sham-operated animals and 7.7 ± 0.4 Hz (*n* = 10; *P* < 0.001) in SNI animals], and the extent of EPSC potentiation remained similar to that seen in the female WT (Orai1*^fl/fl^*) counterparts (Fig. 5, H and I). Thus, in female mice, deletion of microglial Orai1 does not dampen the maladaptive synaptic potentiation in response to nerve injury.

Because changes in inhibitory synaptic transmission are also thought to contribute to chronic pain (*4*, *8*), we next examined whether Orai1 modulates inhibitory synaptic transmission following nerve injury. In contrast to the changes in excitatory synaptic transmission, we did not observe alterations in the frequency of spontaneous inhibitory synaptic currents (sIPSCs; fig. S6), nor were there any changes in the amplitude of the IPSCs following SNI (fig. S6). Likewise, SNI did not induce alterations in the amplitude or frequency of the miniature IPSCs (fig. S7). Furthermore, no differences in either frequency or amplitude were seen between the WT and Orai1 cKO groups (fig. S7). Thus, under our experimental conditions, SNI did not elicit changes in the frequency or amplitude of inhibitory synaptic transmission in lamina II neurons in the mouse spinal cord.

To further assess the mechanisms of synaptic potentiation by SNI and the contributions of microglial Orai1 channels to this process, we carried out measurements of miniature EPSCs (mEPSCs) in the absence of action potentials to address potential changes in the presynaptic release probability (fig. S8). As shown previously (*51*), we found that in the presence of 1 μM tetrodotoxin (TTX) to block action potentials, SNI-treated mice showed significant increases in the frequency of mEPSCs (fig. S8C). Analysis of the miniature events revealed shortening of the interevent intervals (fig. S8), indicating that nerve injury enhances the rate of glutamate release from excitatory lamina II neurons. Male *Orai1^CX3CR1-Cre/ERT2^* mice with deletion of Orai1 in microglia showed smaller enhancement in the mEPSC frequency and shortening of the interevent intervals compared to WT controls (fig. S8, C and D). As seen for spontaneous EPSCs, this phenotype in the mEPSC frequency was only seen in male mice. In female mice, there was no effect of ablating Orai1 on the mEPSC frequency or interevent intervals (fig. S8, H and I).

Together, these results indicate that Orai1 channels in microglia at least partially drive the SNI-mediated increase in presynaptic glutamate release probability in male mice, which contributes to the overall increase in excitatory synaptic transmission in the spinal cord following nerve injury. In female mice, by contrast, loss of microglia Orai1 channels has no effect in mitigating the maladaptive potentiation of excitatory synaptic potentiation seen following nerve injury. Thus, the contribution of microglial Orai1 channels to the potentiation of excitatory synaptic transmission in response to nerve injury evidently differs between male and female mice.

### Orai1 regulates SNI-induced microglial proliferation in the lumbar spinal cord of male mice

**Fig. 6.**
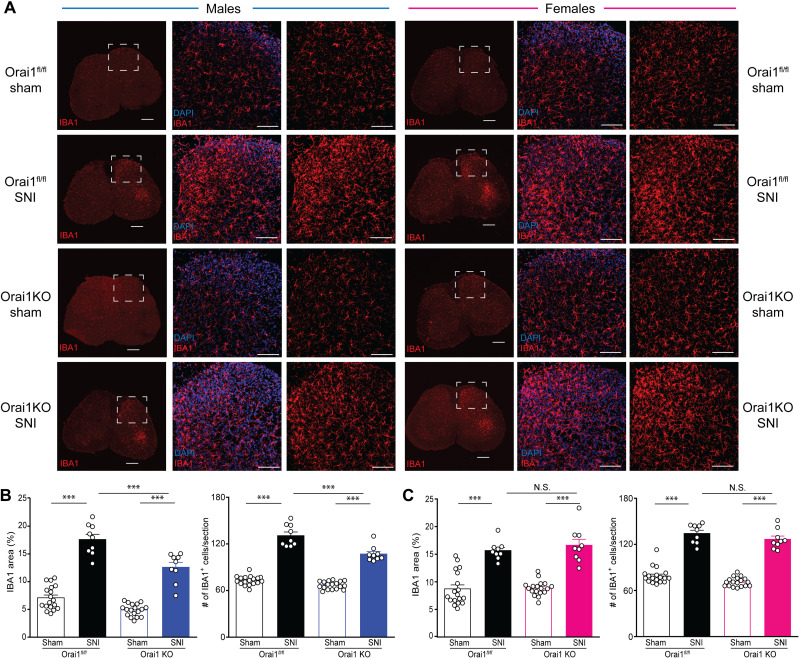
Microglial proliferation is impaired in male but not female Orai1 cKO mice after nerve injury. (**A**) IBA1 staining of transverse sections of the lumbar spinal cord. The images in the first column (for males and females, respectively) show fixed sections of the spinal cord stained for IBA1. Scale bars, 250 μm. Images in the middle column show enlarged L4-L5 sections of the dorsal horn labeled with IBA1 (red) and DAPI (blue). The images in the right column show the same sections with only IBA1 labeling. Scale bars, 100 μm. Imaging parameters and display settings (image brightness and gray scale values) were kept constant for all images. (**B** and **C**) Quantification of IBA1 expression in male and female mice determined by measuring the % area of the region of interest (ROI) occupied by the IBA1 fluorescence signal. The right panels in each case show the number of IBA1-positive cells per section obtained by analysis of the colocalization of the IBA1 and DAPI fluorescence images. Data are given as means ± SEM for *n* = 3 images per region from three mice per group. (***P < 0.001 by two-way ANOVA followed by Tukey test between the indicated groups).

The finding that Orai1 deletion strongly inhibits the induction of IL-6, TNFα, and other mediators linked to neuropathic pain led us to consider the implications in vivo for microgliosis in the spinal cord following nerve injury. A widely used approach for examining microglial reactivity and proliferation is by assessing the expression of the microglial marker, IBA1, which is known to markedly increase in the spinal cord following nerve injury (*12*, *16*). In line with these findings, we also observed that IBA1 staining significantly increased in the dorsal horn of L4-L5 spinal cord sections of WT mice following SNI (Fig. 6A). There was no statistically significant sex difference in the degree of IBA1 up-regulation between male and female mice (*P* = 0.54137 comparing WT SNI male versus WT SNI female groups; Fig. 6, B and C), consistent with previous findings that microglial proliferation is comparable following SNI in both sexes (*16*). The increase in IBA1 staining was specific to the L4-5 spinal cord region but did not occur in spinal sections (e.g., T13) away from the sciatic nerve innervation site, indicating that SNI of the sciatic nerve selectively increases microglial reactivity only in those regions of the spinal cord innervated by the injured nerve (fig S9). SNI also caused notable changes in the morphology of spinal microglia in the affected sections: whereas microglia from sham-operated controls showed a ramified appearance with long processes and small cell bodies, microglia in SNI-operated animals adopted swollen cell bodies with short processes more consistent with activated microglia (fig. S9B). Although male Orai1 cKO mice also showed up-regulation of IBA1 labeling following SNI, the degree of IBA1 up-regulation was significantly muted in the Orai1 cKO mice compared to WT counterparts, as assessed both in measures of IBA1 fluorescence area and by the number of IBA1^+^ cells (Fig. 6, A and B). SNI-treated microglia from Orai1-cKO mice also qualitatively exhibited a smaller extent of morphological change compared to cells in sham-operated WT controls (fig. S9B). These results indicate that deletion of Orai1 impairs microgliosis in vivo in male mice following nerve injury. By contrast, female mice showed no quantitative difference in IBA1 labeling between Orai1 KO and WT groups both in the measurements of IBA1 area and in the number of IBA1-positive cells (Fig. 6, A and C). Thus, deletion of Orai1 does not affect microglial proliferation in female mice. The lack of effect on microglial reactivity parallels the lack of effects on spinal cytokine levels (Fig. 4), neuropathic pain measures (Fig. 3), and degree of synaptic potentiation (Fig. 5) in the female mice, suggesting a direct link between the differential effects of Orai1 on microgliosis and downstream effector phenotypes in male and female mice. Together, these results indicate that Orai1 deletion impairs microglial activation and proliferation in male but not female mice.

Is the up-regulation in IBA1 labeling induced by SNI due to altered Orai1 expression? Despite the evidence for involvement of Orai1 in mitigating pain hypersensitivity and reducing microglial reactivity in male mice, RNA sequencing (RNA-seq) analysis of the lumbar spinal cord (*52*) indicated that SNI did not evoke changes in the expression of Orai1, nor in the expression of other SOCE molecules including Orai2, STIM1, and STIM2 (fig. S10). Similarly, no change in expression of the SOCE transcriptome was detected in female mice (fig. S10). Thus, the most straightforward interpretation is that Orai1-mediated microgliosis in male mice following SNI is not caused by changes in Orai1 expression but instead is likely mediated by the activity of pre-existing microglial Orai1 channels.

### An Orai1 channel blocker mitigates allodynia in male but not female mice

The finding that male microglial Orai1 KO mice show attenuation of mechanical hypersensitivity following nerve injury led us to next consider whether pharmacological suppression of Orai1 can offer similar protection. A previous study showed that systemic delivery of the Orai1 channel antagonist, BTP2, decreases thermal and mechanical hypersensitivity following nerve injury in C57BL/6 mice (only male mice were used in that study) (*53*). However, because BTP2 has also been shown to affect the activity of other ion channels, most notably, transient receptor potential melastatin 4 (TRPM4) (*54*) and ryanodine receptors (*55*), the specificity of this effect is not well resolved. We therefore sought to examine the effects of a more selective Orai1 antagonist, CM4620, which strongly inhibits SOCE in primary cultured spinal microglia (fig. S2A). CM4620 is currently in human clinical trials for mitigating inflammation in acute pancreatitis (*33*) and in patients with coronavirus disease 2019 (COVID-19) pneumonia (*34*). CM4620 shows greater selectivity for Orai1 than Orai2 or Orai3 (fig. S11) and readily accumulates in the brain (see Materials and Methods). We administered CM4620 (or vehicle) by oral gavage to WT C57BL/6 mice for 7 days beginning with the day of the SNI surgery and assessed neuropathic pain due to nerve injury by measuring paw withdrawal thresholds to von Frey filaments as described earlier.

**Fig. 7.**
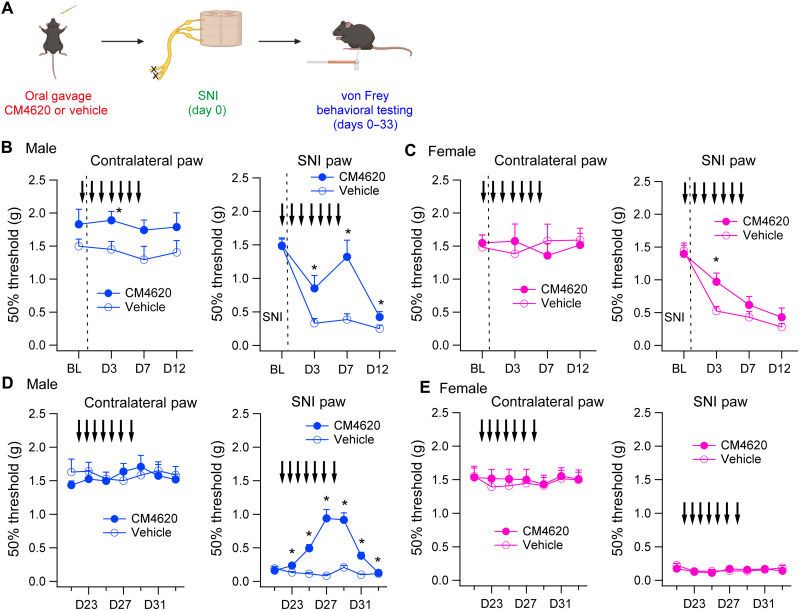
The Orai1 antagonist, CM4620, mitigates SNI-induced mechanical hypersensitivity. (**A**) A schematic of the experiment. Eight- to 10-week-old WT (*Orai1^fl/fl^*) mice were subjected to SNI (on the day indicated by the dotted line), and CM4620 (10 mg/kg) was delivered by oral gavage for 7 days as indicated by the arrows. von Frey thresholds were determined at periodic intervals for 12 days after SNI. The mice were then rested for 9 days and retested beginning day 21. (**B** and **C**) von Frey thresholds in male (B) or female (C) mice before and after SNI surgery in WT (*Orai1^fl/fl^* mice) administered with either CM4620 or just the vehicle. The dotted line indicates the day of SNI surgery. (**D** and **E**) On day 21 following the SNI procedure, a new set of baseline values were collected from the same mice, and CM4620 or vehicle control was delivered for 7 days from the next day as indicated. *n* = 8 mice, CM4620 group; *n* = 7 mice, vehicle group. *P < 0.05 by unpaired *t* test for comparison between CM4620 and vehicle groups.

We found that male WT mice that received the drug were protected for mechanical hypersensitivity on days 3 to 7 following the SNI procedure (Fig. 7B). Furthermore, in male mice, the mitigation of allodynia only lasted for the duration of the drug administration. Cessation of the drug on day 7 caused the paw withdrawal thresholds in WT mice to return to levels seen in control mice by day 12 (Fig. 7B, right graph). This result suggests that CM4620 fundamentally does not block the induction of neuropathic pain but instead reverses the pain hypersensitivity. Female mice did not show attenuation of allodynia relative to the vehicle control group (Fig. 7C). To directly determine whether CM4620 is also effective in relieving allodynia once it is already induced, we examined the effects of readministering the drug on day 22 after SNI (after collecting a new baseline the prior day; Fig. 7D). Administering CM4620 on day 22 after the induction of mechanical allodynia also relieved hypersensitivity in male mice receiving the drug (Fig. 7D, right graph). As before, the pain relief subsided quickly (~2 days) following cessation of the drug, similar to the rapid development of allodynia when the drug was withdrawn after nerve injury (Fig. 7B). Female mice, however, did not show any attenuation of the hypersensitivity under any of these conditions (Fig. 7E).

If the effect of CM4620 is mediated by inhibition of Orai1 in microglia, then one predicts that this effect would be occluded in Orai1 cKO mice. To test this prediction, we next administered CM4620 in male Orai1 cKO mice to determine whether delivery of the drug elicits additional analgesia compared to the protection seen in Orai1 cKO mice alone (fig. S12). Over a duration of 31 days, these experiments indicated that neither during the first few days after nerve injury nor starting 21 days after the injury was there an effect of CM4620 in the Orai1 cKO mice (fig. S12). One time point (day 7) following SNI surgery did show enhanced sensitivity in the mice getting CM4620; however, there was no further effect in the remaining time points. Thus, the effect of the drug depended on the presence of the drug target in microglia. We interpret the lack of added protection in the mice getting the drug as indicative that the analgesic effect of CM4620 is likely directly due to inhibition of Orai1 in pain pathways mediated by microglia rather than by other cell types.

Together, these results indicate that in vivo delivery of an Orai1 antagonist mitigates mechanical hypersensitivity in the SNI model of neuropathic pain, confirming the genetic validation of microglial Orai1 as a target for neuropathic pain (Fig. 3). Moreover, the sex-specific suppression of allodynia in male but not female mice getting the drug matches the phenotype of female microglial Orai1 cKO mice, suggesting that CM4620 inhibition of microglial function may underlie the sex-specific amelioration of neuropathic pain in the WT mice. The ability of CM4620 to reverse hypersensitivity 3 weeks after the induction of SNI indicates that Orai1 blockers may hold promise for the development of a new line of therapeutics against neuropathic pain.

## DISCUSSION

There is strong interest in understanding the microglial checkpoints regulating neuroinflammation and several signaling cascades involving the P2X and P2Y receptors, and p38 MAP kinase has received much attention in activating spinal microglia following nerve injury (*2*, *4*). Orai1 channels are a well-known molecular checkpoint for inflammatory processes in many immune cells (*24*, *39*), but their role in microglia-mediated neuroinflammation that underlies neuropathic pain has not been explored. In this study, we show that Orai1 channels are a major mechanism for agonist-evoked Ca^2+^ signals in microglia, and their activation stimulates synthesis and release of key inflammatory microglial mediators. Conditional deletion of Orai1 in microglia attenuated microglial proliferation, the production of proinflammatory spinal cytokines, and the maladaptive potentiation of excitatory synaptic transmission in the spinal dorsal horn. Unexpectedly, these effects are seen only in male mice, and female microglial Orai1 KO mice show no differences in these cellular and physiological end points. Furthermore, behavioral analysis revealed that male, but not female, microglial Orai1 KO mice show partial protection against mechanical hypersensitivity following nerve injury, mirroring the cellular phenotypes. Together, these results identify Orai1 channels as critical mediators of microglia-mediated neuroinflammation and the sex-specific effects of microglia for neuropathic pain.

Our Ca^2+^ imaging experiments indicate that Orai1 is needed for mediating SOCE in spinal microglia (Fig. 1), indicating that Orai1 is an essential component of the microglial Ca^2+^ signaling toolkit (*23*, *56*, *57*). Furthermore, deletion of Orai1 abrogated Ca^2+^ influx arising from stimulation of microglial ATP receptors including the P2Y6 and P2Y12 receptors. This is significant as ATP that is released from hyperexcited (or damaged) neurons and other cells in the spinal cord, signaling via P2X and P2Y receptors on microglia, is a major driver of neuroinflammation and induction of neuropathic pain (*5*, *7*). In line with this hypothesis, behavioral phenotyping revealed that male *Orai1^fl/fl Cx3CR1-Cre/ERT2^* mice are partially protected against the induction of mechanical allodynia following SNI. Protection in these mice was robust and sustained, lasting >2 weeks following nerve injury (Fig. 3). Likewise, mitigation of SNI-induced pain hypersensitivity was replicated by CM4620, a small-molecule Orai1 antagonist that is currently in clinical trials for mitigating inflammation in human patients with acute pancreatitis and COVID-19 pneumonia (*33*, *34*). Thus, two orthogonal lines of evidence (cKO and drug) converge on the same male-specific effect. CM4620 lost its protective effect in Orai1 cKO mice, demonstrating that the drug’s effects converge on the same cellular pain pathways mediated by Orai1 in microglia. Cessation of CM4620 resulted in the reemergence of mechanical hypersensitivity, suggesting that acute blockade of Orai1 function does not prevent the induction of neuropathic pain but rather only its continued maintenance.

The selective effects of Orai1 deletion on spinal cytokines in male but not female mice is reminiscent of the past observations that broad spectrum inhibitors of glial activation by tetracycline antibiotics, ablation of microglia with toxins, or inhibition of microglial TLR4 or p38 signaling only protect male but not female mice in models of neuropathic pain (*11*, *12*, *15*, *16*, *37*). In addition to illustrating another notable example of a specific genetic perturbation yielding markedly distinct pain phenotypes in males and females, our results also bear on two key questions that are of strong interest. First, our results suggest that the sex difference in neuropathic pain in male and female Orai1 cKO mice is directly explained by differences in proinflammatory cytokines. In male Orai1 cKO mice, the SNI-induced enhancement of IL-6 was completely blocked, and the increases in TNFα, IL1-β, and BDNF in the spinal cord were cut in half compared to WT counterparts (Fig. 4). In female mice, by contrast, the enhancement of these inflammatory mediators by SNI was not affected by the deletion of Orai1 in microglia. Thus, the behavioral sex difference in pain hypersensitivity is well correlated with inflammatory cytokine changes in the spinal cord. Second, our results indicate that male and female mice with differential pain regulation show differences in synaptic potentiation following nerve injury. In response to conditional deletion of Orai1 in microglia, the maladaptive potentiation of excitatory synaptic transmission following SNI is partially blocked in male but not female mice (Fig. 5). The potentiation of excitatory synaptic transmission is correlated with the changes in proinflammatory spinal cytokines following nerve injury. Thus, these results trace a direct link between the sex differences in enhancement of spinal inflammatory cytokines levels with the pathological synaptic potentiation and pain hypersensitivity following nerve injury.

Two lines of evidence indicate that the lack of change of the up-regulation of cytokine levels, excitatory synaptic enhancement, and allodynia in female *Orai1^fl/fl Cx3CR1-Cre/ERT2^* mice following SNI is not because Orai1 is dispensable for the physiology of female microglia. First, deletion of Orai1 in the isolated female microglia abrogated SOCE equally from isolated microglia in both males and females (fig. S2). This finding indicates that Orai1 is essential for agonist-evoked SOCE in both males and females. Second, deletion of Orai1 impaired cytokine synthesis (IL-6, TNFα, MCP1, and PGE_2_) from primary cultures of microglia in both males and females (Fig. 2), indicating that the difference between males and females in the cytokine synthesis is not a cell-autonomous feature of microglia but likely due to extrinsic influences in vivo. What then could be the basis of the in vivo male-female difference in the microglial Orai1 cKO mice? We suggest that the behavioral differences in pain mitigation between the sexes and effects on spinal cytokines and synaptic potentiation are a direct consequence of differential microglial proliferation in Orai1 cKO mice following SNI (Fig. 6), which is Orai1 dependent in males but not females. Recent evidence indicates that female mice show greater dependence on adaptive immune cells (T cells) relative to male mice and are protected against neuropathic pain only when T cells and microglia are both depleted (*12*). Microglia and T cells can interact in numerous ways in the CNS including cell-to-cell contact and cytokine-mediated communication to promote neuroinflammation (*58*). We postulate that female mice likely use hormonally regulated adaptive immune cell (T cell) signaling to support allodynia through T cell–microglial interactions. These interactions must use a non-Orai1 pathway because CM4620 was ineffective in female mice. Males, on the other hand, rely on an Orai1 channel–dependent pathway in microglia. Additional studies using in vivo two-photon Ca^2+^ imaging may reveal differences in microglial Ca^2+^ signaling between the sexes following SNI and shed light on these questions, but the results presented here provide a framework for evaluating these and other hypotheses.

## MATERIALS AND METHODS

### Transgenic mice

Male and female C57BL/6 mice were used in this study and cared for in accordance with institutional guidelines and the *Guide for the Care and Use of Laboratory Animals*. All animals were group-housed in a sterile ventilated facility, under standard housing conditions (12-hour light/12-hour dark cycle with lights on at 7:00 a.m. and temperatures of 20° to 22°C with ad libitum access to water and food), and maintained with in-house breeding colonies. Male and female mice were used in approximately equal numbers. All research protocols were approved by the Northwestern University Institutional Animal Care and Use Committee. The *Orai1^fl/fl^* mice have been described previously (*32*). Tissue-specific deletion of Orai1 in microglia was accomplished by crossing *Orai1^fl/fl^* mice with *Cx3CR1-Cre/ERT2* mice (the Jackson Laboratory) to generate *Orai1^fl/fl Cx3CR1-Cre/ERT2^* mice. Cre recombinase activity was induced by repeated tamoxifen injections. Briefly, 4- to 5-week-old mice were injected intraperitoneally for 5 days with tamoxifen (1 mg/kg; T5648, Sigma-Aldrich, USA) dissolved in 200 μl of corn oil (C8267, Sigma-Aldrich) for 5 days. Alternately, in some experiments (Figs. 4 and 5), mice were dosed with tamoxifen (8 mg/kg) twice (48 hours apart). To delete Orai1 in vitro, OH-tamoxifen (H7904, Sigma-Aldrich, USA) was delivered to the cells at a final concentration of 1 μM to 25 cm^2^ directly into the culture flasks at least 3 days before harvesting microglia. Ethanol was used as a solvent control for the in vitro experiments.

### Primary cultures

Primary microglia were isolated from neonatal (P0 to P3) mice from astrocyte-glial cultures, which were grown as previously described for hippocampal tissue (*28*). Briefly, spinal cord was dissected, and meninges were removed under a dissection microscope in 4°C dissection medium [10 mM Hepes in Hanks’ balanced salt solution (HBSS)]. After enzymatic dissociation with 0.25% trypsin (Invitrogen, Q10602319) and deoxyribonuclease (DNase; 1 mg/ml; Roche Diagnostics, 10104159001) for 15 min in a 37°C water bath, tissue was washed twice with HBSS (Gibco, 14175-095) and dissociated gently by trituration in culture media consisting of Dulbecco’s modified Eagle’s medium (DMEM; Corning, 10-013-CM) with 10% fetal bovine serum (Gibco, 10082-139) and 1% penicillin-streptomycin (pen-strep; Gibco, 15140122) solution. Dissociated cells were filtered through a 70-μm strainer to collect cell suspension and cultured in 25-mm^2^ tissue culture flasks on poly-L-lysine–coated (P4707, Sigma-Aldrich, Schnelldorf, Germany) with 10 ml of medium. When cultures were made for Ca^2+^ imaging experiments to evaluate SOCE differences in males and females, cells were cultured separately from each pup. The spinal cord was dissected, and cells were isolated, as described above, and cultured in T25 flask for 2 weeks. For ELISA experiments evaluating cytokine production in cultured microglia (Fig. 2), two spinal cords from neonatal mice of the same sex were used per flask. Half of the medium was exchanged every 3 to 4 days until cells reached near confluence (7 to 10 days in vitro). Microglia were collected by forcefully shaking by hand for 1 min or by shaking cells in an incubator for 1 hour at 200 rpm and centrifuged for 5 min. The supernatant was removed, and the cell pellet was resuspended in culture medium. Cells were plated on poly-d-lysine–coated glass-bottom dishes (14 mm in diameter; 10,000 to 15,000 cells per coverslip; MatTek) or 24-well plates (15,000 to 20,000 cells per well). Microglia were maintained in the incubator and used 1 week in culture for the Ca^2+^ imaging and ELISA studies and 2 weeks for the experiments shown in fig. S2.

### Isolation of microglia from adult mice

A Percoll gradient was used for isolation of adult microglia in mice as previously described (*59*). Briefly, 12-week-old mice were anesthetized with isoflurane and perfused intracardially with cold phosphate-buffered saline (PBS). Brain and spinal cords were extracted from each mouse and minced in DMEM/F12 (Corning, 10-090-CV) with 1% pen-strep and 1% glucose. Minced tissue was then digested in DMEM/F12 supplemented with DNase I (20 U/ml; Roche Diagnostics, 10104159001), papain (1 mg/ml; Worthington Biochemical Corporation, LK003178), and dispase II (1.2 U/ml; Millipore Sigma, D4693-1G) at 37°C for 20 min. Afterward, the dissociation was treated with DMEM/F12 with 10% heat-inactivated fetal bovine serum and 1% pen-strep and washed twice with DMEM/F12 supplemented with 1% glucose and 1% pen-strep. Tissue was mechanically dissociated by pipetting up and down with a P1000 pipette tip. Dissociated tissue was collected and transferred to a new tube. The dissociation step was repeated with fresh media and then a final time with a P200 pipette tip. The collected cells from the tissue dissociation procedure were filtered through a 70-μm cell strainer and centrifuged. Supernatant was removed, and the cell pellet was resuspended in 37% stock isotonic Percoll (SIP; 3.7 ml of SIP solution and 5.3 ml of 1× HBSS). SIP solution was one part 10× HBSS (Gibco, 14185-052) and nine parts Percoll (Sigma-Aldrich, P493). A 70% SIP layer was slowly added below the 37% SIP cell–containing layer, and the 30% SIP solution was added above the 37% SIP layer. Last, 2 ml of 1× HBSS was added above the 30% SIP layer and centrifuged for 40 min at 300*g* and 18°C with no brake. After centrifugation, 2 ml of the solution was collected at the 70 to 37% interphase layer and transferred to a new tube. This interphase layer was washed with 6 ml of 1× HBSS and centrifuged at 500*g* for 7 min. The cell pellet was resuspended in 500 μl of HBSS and transferred to a 1.5-ml tube followed by washing three times with 1× HBSS and used for RNA extraction and qRT-PCR.

### Ca^2+^ imaging

Primary spinal microglia grown on poly-d-lysine–coated glass-bottom dishes were loaded with Fura-2 by incubating cells in 2 μM Fura-2-AM (Invitrogen, F1221) in growth medium for 30 min at 37°C. Fura-2–containing medium was washed off, and cells were incubated for an additional 10 min before imaging. All experiments were performed at room temperature. Single-cell [Ca^2+^]_i_ measurements were performed as described previously (*28*). Image acquisition and analysis were performed using SlideBook (Denver, CO). Dishes were mounted on the stage on Olympus IX71 inverted microscope, and images were acquired every 6 s at excitation wavelengths of 340 and 380 nm and an emission wavelength of 510 nm. For data analysis, regions of interest (ROIs) were drawn around single cells, background was subtracted, and *F*_340_/*F*_380_ ratios were calculated for each time point.

[Ca^2+^]_i_ was estimated from *F*_340_/*F*_380_ ratio using the standard equation: [Ca^2+^]_i_ = β*K*_d_ (*R* − *R*_min_)/(*R*_max_ − R), where *R* is the *F*_340_*/F*_380_ fluorescence ratio and values of *R*_min_ and *R*_max_ were determined from an in vitro calibration of Fura-2 pentapotassium salt. β was determined from the *F*_340_/*F*_380_ ratio at 380 nm, and *K*_d_ is the apparent dissociation constant of Fura-2 binding to Ca^2+^ (135 nM). For each cell, the rate of SOCE (Δ[Ca^2+^]_i_/Δ*t*) was calculated from the slope of a line fitted to three points (18 s) after the readdition of 2 mM Ca^2+^_o_. Baseline [Ca^2+^]_i_ was calculated by averaging [Ca^2+^]_i_ values over 2-min baseline for each experiment. Store release was calculated by measuring the area under the curve during TG application in Ca^2+^-free solution. The standard Ringer’s solution used for these experiments contained the following: 155 mM NaCl, 4.5 mM KCl, 10 mM d-glucose, 5 mM Hepes, 1 mM MgCl_2_, and 2 mM CaCl_2_. The Ca^2+^-free Ringer’s solution was similar to the above solution except that it contained 3 mM MgCl_2_ and 1 mM EGTA (Sigma-Aldrich) with no added CaCl_2_. pH was adjusted to 7.4 with 1 M NaOH. Stock solution of TG was dissolved in dimethyl sulfoxide (DMSO) and used at the indicated concentration.

### Immunostaining of cultured microglia

Isolated microglia were seeded on poly-d-lysine–coated glass coverslips and fixed at room temperature using 4% paraformaldehyde (PFA) for 15 min. Cells were then washed twice with PBS, permeabilized, and blocked using 4% bovine serum albumin (BSA)/4% normal goat serum/0.1% Triton X-100 for 1 hour. Cells were then washed twice using PBS and incubated overnight with 1:1000 IBA1 antibody (Novus, NBP2-19019) in PBS/4% BSA at 4°C. The next day, cells were washed with PBS containing 0.1% Tween 20 and then incubated by secondary goat rabbit Alexa Fluor 488 antibody (Thermo Fisher Scientific; 1:1000 in PBS/4% BSA) at room temperature in the dark. Cells were then washed three times with PBS dissolved with 0.1% Tween 20 and mounted using ProLong Gold Antifade Mount medium with 4′,6-diamidino-2-phenylindole (DAPI; Invitrogen, P36931). Microglia were imaged on an upright Nikon A1R confocal microscope.

### Quantitative RT-PCR

Microglia cultures from mouse pup spinal cords and isolation of adult microglia from brain and spinal cord were lysed for total RNA extractions using the RNeasy Plus Mini Kit (74134, QIAGEN). cDNA generation was performed using the High-Capacity cDNA Reverse Transcription Kit (4368814, Applied Biosystems by Thermo Fisher Scientific). PowerUp SYBR Green Master mix (A25741, Applied Biosystems by Thermo Fisher Scientific) was used for qPCR and followed according to the manufacturer’s instructions. For qPCR, each well contained 7 ng of cDNA and final primer concentration of 500 nM. The qPCR was run using the CFX Connect Real-Time System from Bio-Rad, and data were analyzed using the Bio-Rad CFX Maestro software. Sequences of primers were as follows: Gapdh forward, 5′-AGGTCGGTGTGAACGGATTTG-3′; Gapdh reverse, 5′-ATGTA GACCATGTAGTTGAGGTCA-3′;18*S* forward, 5′-TGCGAGTACTCAACACCA ACA-3′; 18*S* reverse, 5′-CTGCTTTCCTCAACACCACA-3′; Orai1 forward, 5′-AGACTGCCTGATCGGATGGC-3′; Orai1 reverse, 5′-TTGTCCCCGAGCCATTTCCT-3′; Orai2 forward, 5′-GCAGCTACCTGGAACTCGTC-3′; Orai2 reverse, 5′-GTTGTGGATGTTGCTCCCG-3′; Orai3 forward, 5′-CAGTCAGCACTCTCTGCGG-3′; Orai3 reverse, 5′-TGGCCACCATGGCGAAG-3′; Stim1 forward, 5′-ATTCGGCAAAACTCTGCTTC-3′; Stim1 reverse, 5′-GGCCAGAGTCTCAGC-3′; Stim2 forward, 5′-TCGAAGTGGACGAGA-3′; and Stim2 reverse, 5′-TTTCCACTGTTTCCAC-3′.

### ELISA analysis

For analysis of cytokine levels in spinal tissue in vivo, the spinal cord (5 mm in length) at the lesion epicenter was dissected out from each animal (five animals per group) 7 days after SNI surgery. Spinal cord tissue was homogenized by mechanical trituration followed by sonication in radioimmunoprecipitation assay lysis buffer (1 ml; Santa Cruz Biotechnology Inc., Santa Cruz, CA, USA), and the cytokine concentration was measured using mouse ELISA kits (Invitrogen Life Technologies, Carlsbad, CA, USA) according to the manufacturer’s instructions. Cytokine levels were expressed in pg/mg.

For the in vitro cytokine measurements from cultured cells, primary microglia isolated from spinal cords were seeded into 24-well plates at a density of 20,000 cells per well. Serum was removed from the media 24 hours before treatments were added to the wells. Cells were treated with DMSO, LPS (1 ng/ml; Sigma-Aldrich, L4391), or TG (0.25 μM; Sigma-Aldrich, T9033) and PDBu (25 nM; Sigma-Aldrich, P1269) for 18 hours. Supernatants were subsequently analyzed using mouse ELISA kits for TNFα (R&D Systems, catalog no. MTA00B), IL-6 (RayBiotech, catalog no. ELM-IL6-1), PGE_2_ (Cayman Chemical, catalog no. 514010), and MCP1 (RayBiotech, catalog no. ELM-MCP1-CL-5). All kits were used according to the manufacturers’ instructions. Release of proinflammatory factors is expressed in concentration of pg/ml.

### Preparation of spinal cord slices

Spinal cord slices obtained from mice at 7 days after SNI surgery were used for electrophysiological assessments. The mice were deeply anesthetized with isoflurane and then subjected to a rapid intracardiac infusion of ice-cold oxygenated high-sucrose artificial cerebrospinal fluid (sACSF) containing 95 mM NaCl, 1.8 mM KCl, 1.2 mM KH_2_PO_4_, 0.7 mM CaCl_2_, 1 mM MgCl_2_, 26 mM NaHCO_3_, 50 mM sucrose, and 15 mM d-glucose. The lumber segments (L3 to L5) were quickly removed and immersed in ice-cold oxygenated sACSF. The pH was adjusted to pH 7.4 (osmolality, 300 to 310 mosm), and the fluid was oxygenated with 95% O_2_ and 5% CO_2_. To identify the ipsilateral side, a sharp knife was used to make a deep mark in the contralateral side. The spinal cord was then transversely sliced into 400-μm sections using a tissue slicer (Compresstome model VF-200-0Z, Precisionary Instruments). Slices were transferred to a recovery ACSF chamber containing 125 mM NaCl, 2.4 mM KCl, 1.2 mM Na_2_PO_4_, 1 mM CaCl_2_, 2 mM MgCl_2_, 25 mM NaHCO_3_, and 25 mM d-glucose, while the slices were maintained at 30°C. Individual slices were transferred to a recording chamber and perfused with normal ACSF containing 2 mM CaCl_2_.

### Slice electrophysiology

The lamina II (SG) in the superficial dorsal horn was visually identified as translucent wide band of tissue in spinal slices under differential interference contrast imaging. EPSCs and IPSCs were recorded from lamina II neurons in the whole-cell patch-clamp technique with an Axopatch 200B amplifier interfaced to an ITC-18 input-output board and iMac G5 computer. Currents were filtered at 2 kHz with a four-pole Bessel filter and sampled at 5 kHz. Data acquisition and analysis were performed using in-house routines developed on the Igor Pro platform. Voltage-clamped recordings were performed in the whole-cell recording mode with a pipette potential of −70 mV. Patch electrodes were pulled from borosilicate capillary glass using a vertical pipette puller and had a resistance of 4 to 6 megohm. The pipettes were filled with an intracellular solution containing 135 mM K-gluconate, 5 mM KCl, 5 mM EGTA, 5 mM Mg-ATP, 5 mM Hepes, 0.5 mM CaCl_2_, and 2 mM MgCl_2_ (adjusted to pH 7.2 with tris base) for recording of EPSCs or 95 mM CsF, 25 mM CsCl, 10 mM Hepes, 10 mM EGTA, 2 mM Mg-ATP, 0.3 mM Na_3_-guanosine triphosphate, 10 mM QX-314, 5 mM tetraethylammonium chloride, and 5 mM 4-aminopyridine (pH 7.3 with KOH or CsOH) for IPSCs. Spontaneous EPSCs were recorded in the presence of picrotoxin (50 μM) and d-(-)-2-amino-5-phosphonopentanoic acid (D-APV) (50 μM). sIPSCs were isolated by administering 6-cyano-7-nitroquinoxaline-2,3-dione (CNQX) (10 μM) and D-APV (50 μM) to the slices in the extracellular solutions. mEPSCs and miniature IPSCs (mIPSCs) were recorded in the presence of TTX (1 μM).

### Immunohistochemistry of spinal cord slices

Mice were anesthetized with isoflurane and perfused intracardially with cold PBS followed by 10% formalin 14 days after SNI. Spinal cords were extracted, and the lumbar area was isolated and fixed in 10% formalin solution overnight. The next day, spinal cords were placed in 30% sucrose PBS solution. Spinal cords were sectioned at 40-μm thickness. Sections were washed and blocked with 10% goat serum in tris buffered saline (TBS) with 0.3% Triton X-100 for 2 hours at room temperature. Slices were incubated with rabbit anti-IBA1 primary antibody (1:1000; Wako Chemicals USA, catalog no. 019-19741) at 4°C overnight. Sections were washed and incubated with secondary antibody (1:1000; goat anti-rabbit Alexa 594 from Thermo Fisher Scientific) and DAPI for 2 hours at room temperature. Sections were washed and mounted with VECTASHIELD (Vector Labs, H-1200-10). Images of the spinal cord and spinal dorsal horns were acquired using a Nikon A1R confocal microscope with a 25× objective. The images of whole spinal cord were taken by stitching images together and used to identify L4 to L5 area of the spinal cord. If a section had increased IBA1^+^ staining in the ventral horn, then a *z*-stack image was acquired of the dorsal horn. All image acquisition settings were maintained between sexes, genotypes, and injury groups. The number of stacked images and step size was kept the same between sexes, genotypes, and injury groups. Before quantification, the *z*-stack images were flattened to maximum intensity projections and then subsequently processed for percent area of IBA^+^ staining. The background intensity was determined and subtracted from a region that of the slide that did not contain tissue, and the dorsal horn was outlined to create an ROI. This ROI was used to quantify the percent area of IBA1^+^ staining detectable above background. Images were analyzed with NIS Elements software and acquisition parameters (gain, laser power, offset, pixel dwell, pinhole, line averaging, step size, and number of *z*-steps) were kept the same between sexes, genotypes, and injury groups. The number of microglia per section was counted by overlaying a DAPI mask with the IBA1 stain. The number of microglia was manually counted by a blinded experimenter.

### RNA-seq analysis

RNA-seq analysis of spinal tissue from male and female mice is described in a companion study (*52*). Briefly, the L4-L5 lumbar dorsal horn of the spinal cord was harvested on postoperative day 7 to study transcriptional changes in dorsal spinal tissue. RNA was extracted, and transcriptional analysis was done on the basis of comparison to the GRCm38-mm10.0 genome. Genes with adjusted *P* values <0.01 and fold changes greater than 0.5 were defined as differentially expressed genes. A total of 24 samples from mice were analyzed [divided between the Sham-ipsi, Sham-contra, and SNI_ipsi groups; see (*52*)].

### SNI surgery

SNI surgeries were performed following the procedures described previously (*42*). Under anesthesia with isoflurane in O_2_ (induction: 3% isoflurane; maintenance: 2% isoflurane), the left sciatic nerve was exposed after sectioning the skin of the posterior aspect of the thigh and incision through the fat pad covering the popliteal fossa. The common peroneal and the tibial nerves were identified and ligated with 5-0 silk thread and transected distal to the ligation, removing 2- to 4-mm length of each nerve. The sural nerve remained intact, and any contact with or stretching of this nerve was carefully avoided. Muscle and skin were closed in two distinct layers. For sham surgeries, the sciatic nerve trifurcation was only exposed without ligation and transection.

### Mechanical withdrawal threshold assessment

The 50% paw withdrawal threshold of both hind paws was assessed using von Frey filaments as previously described (*41*). Briefly, the animals were placed individually under separate transparent Plexiglass chambers positioned on a wire mesh floor. Approximately 30 min were allowed for habituation. The mice were sequentially applied a series of von Frey filaments (0.02 to 4.0 g; Stoelting, CO, USA) to the lateral plantar surface of the hind paw. Filaments were perpendicularly applied to the lateral plantar surface of the hind paw in either ascending or descending strengths to determine the filament strength closest to the withdrawal threshold. Each filament was applied for a maximum of 2 s at each interval. Brisk withdrawal and hind paw licking were considered positive as nociceptive responses. Given the response pattern and the force of the final filament, 50% response threshold was calculated. Other behavioral changes were also recorded. All tests were conducted between 1 and 4 p.m. by an examiner who was blind to the experiments.

### Patch-clamp analysis of CM4620 blockade of Orai currents

Patch-clamp recordings of STIM1-activated Orai currents were carried out as previously described (*60*). Briefly, human embryonic kidney 293 cells were transfected with the indicated Orai isoform (Orai1, Orai2, or Orai3) together with STIM1. Twenty-four hours following cell transfection, Orai currents were recorded in the whole-cell patch-clamp configuration. The standard extracellular solution contained 130 mM NaCl, 4.5 mM KCl, 20 mM CaCl_2_, 10 mM tetraethylammonium chloride, 10 mM d-glucose, and 5 mM Hepes (pH 7.4 with NaOH). The internal solution contained 135 mM Cs aspartate, 8 mM MgCl_2_, 8 mM Cs-(1,2-Bis(2-aminophenoxy) ethane-N-N-N'-N' tetraacetic acid (Cs-BAPTA), and 10 mM Hepes (pH 7.2 with CsOH). The holding potential was +30 mV. The voltage stimulus consisted of a 100-ms step to −100 mV followed by a 100-ms ramp from −100 to +100 mV applied at 1-s intervals. Orai current was activated by passive depletion of ER Ca^2+^ stores by intracellular dialysis of 8 mM BAPTA, and the indicated concentration of CM4620 was applied as described in fig. S12. All data were corrected for leak currents collected in 100 μM LaCl_3_.

### Drug administration

Stocks of BTP2 (catalog no. 203890; Sigma-Aldrich) and the powdered form of CM4620 (CalciMedica) were dissolved in DMSO and applied at dilutions of 1000-fold. For the behavioral studies in Fig. 7, 40 mg of CM4620 was mixed with and 9.6 ml of vehicle (0.5% methyl cellulose and 1% Tween 80) for 10 mg/kg (assuming 10 ml/kg and 25% loading of CM4620). All treatments were delivered daily to the mice via oral gavage 7 days from the day of surgery as indicated in the figure legend. CNS penetration of CM4620 was confirmed by determining the ratio of compound concentrations (measured by liquid chromatography–tandem mass spectrometry) in brain tissue extracts to those in plasma after oral or intravenous administration. Under these conditions, the brain-to-plasma ratio ranged from 2.0 to 2.6 in both rats and mice and did not accumulate with repeated dosing. For the drug readministration studies in Fig. 7 (D and E) beginning day 22 after injury, CM4620 or vehicle was administered via oral gavage for 7 days.

### Data analysis

All data are expressed as means ± SEM. Statistical analysis was calculated using SigmaPlot version 13.0 (Systat Software) or Origin2021b with a confidence level of 95%, and results with *P* < 0.05 were considered statistically significant (*P < 0.05, **P < 0.01, and ***P < 0.001). For datasets with two groups, statistical analysis was performed with a two-tailed Student unpaired *t* test to compare between groups. If the two groups had unequal variance, then a Welch correction or a Mann-Whitney rank sum test was used. For datasets greater than two groups, two-way analysis of variance (ANOVA) followed by Tukey post hoc test was performed to compare between groups. mEPSCs and mIPSCs were analyzed using Mini Analysis software (Synaptosoft Inc., GA, USA). The threshold for detection of events was set at 5 pA (for EPSCs) or 10 pA (for IPSCs).

## Notes

View/request a protocol for this paper from *Bio-protocol*.
