# Supplementary material for: Regulation of neuropathic pain by microglial Orai1 channels
Source: Sci Adv. 2023 Jan 27;9(4):eade7002. doi: 10.1126/sciadv.ade7002 (PMC9883051; doi:10.1126/sciadv.ade7002)
Supplement: Supplementary file 1 — Figs. S1 to S12 [file sciadv.ade7002_sm.pdf]

Science Advances
AAAS

Supplementary Materials for

Regulation of neuropathic pain by microglial Orai1 channels

Shogo Tsujikawa *et al.*

Corresponding author: Murali Prakriya, m-prakriya@northwestern.edu

Sci. Adv. **9**, eade7002 (2023)
DOI: 10.1126/sciadv.ade7002

This PDF file includes:

Figs. S1 to S12

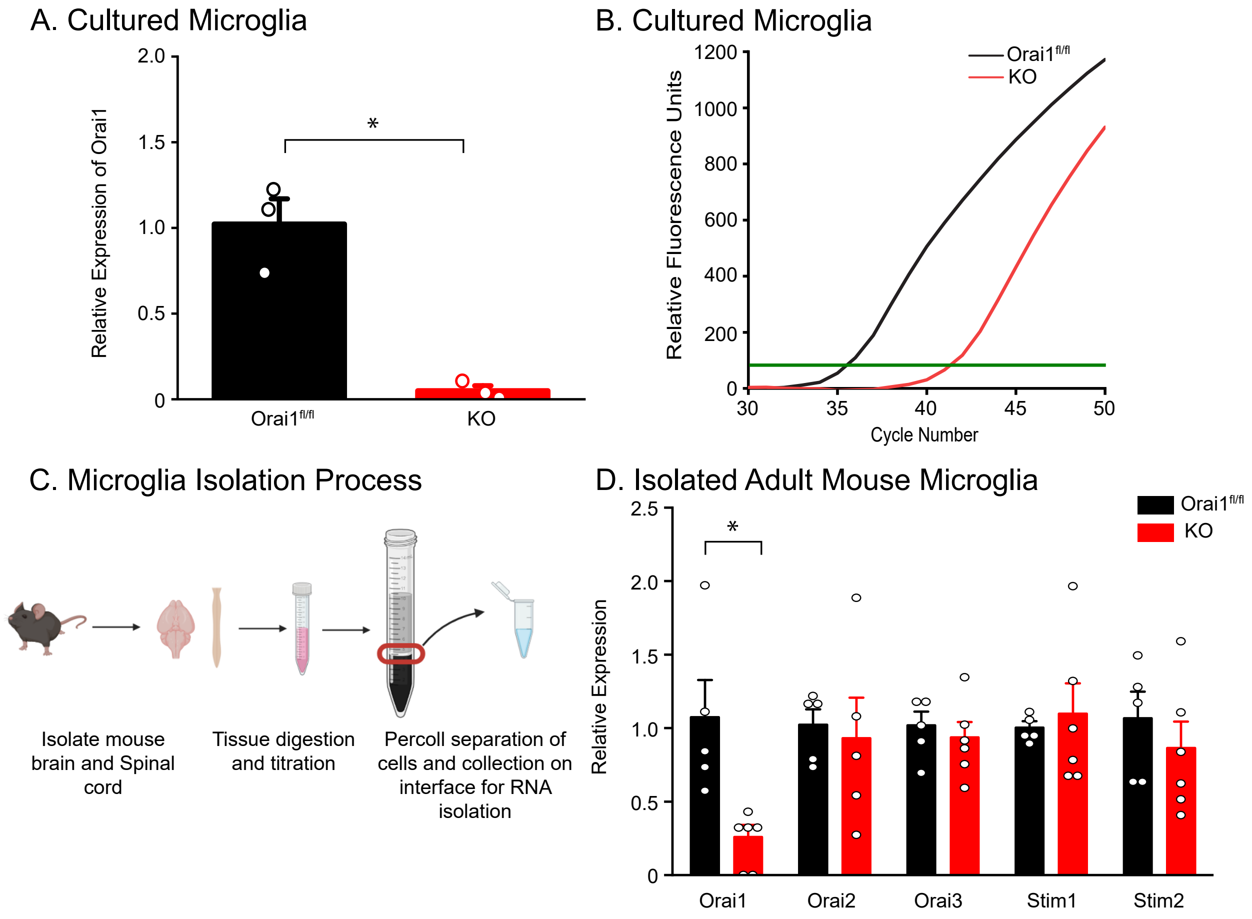

Figure S1. Analysis of Orai1 mRNA expression in primary microglia. Primary microglia were isolated from the spinal cord from P0-P3 pups and Orai1 mRNA was assessed by real-time qPCR. **(A)** Relative expression of Orai1 mRNA in WT (Orai1$^{fl/fl}$) and Orai1 KO (Orai1$^{fl/fl\ CX3CR1\text{-}Cre/ERT2}$) microglia exposed to tamoxifen. mRNA was normalized to GAPDH and 18S mRNA and shown as relative expression. **(B)** Real-time PCR time course of the fluorescence emission of SYBR Green I during amplification of Orai1 cDNA obtained by reverse transcription from mRNA harvested from spinal microglia cultures from WT and Orai1 KO mice. The dashed line denotes noise level. N= 3. P<0.05 by unpaired student's T-test. **(C)** Protocol for isolation of microglia from the adult brain and spinal cord. A Percoll gradient centrifugation step was used to separate adult primary microglia from other cells. **(D)** RT-qPCR analysis of Orai and STIM mRNA from freshly isolated adult microglia. N=5-6. P<0.05 by unpaired student's T-test.

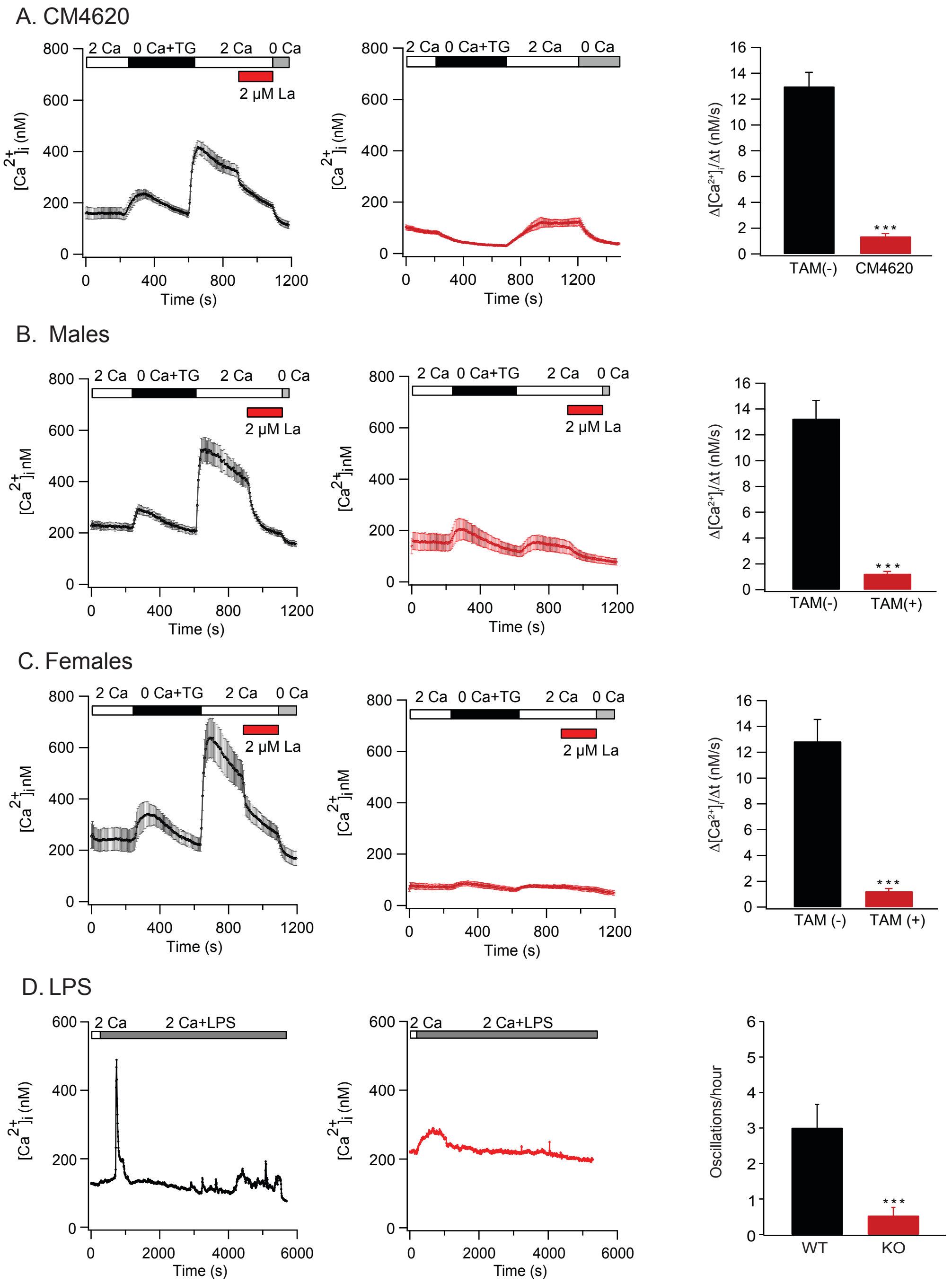

Figure S2. Analysis of SOCE and effects of Orai1 deletion in microglia from male and female mice. (A) Inhibition of SOCE by the Orai1 antagonist, CM4620 in WT (Orai1$^{fl/fl}$) microglia. SOCE was initiated by depleting Ca^{2+} stores with 1μM TG in Ca^{2+} -free medium followed by re-addition of extracellular Ca^{2+} at t= 500 s. The bar graph on the right shows the rate of Ca^{2+} entry following extracellular Ca^{2+} re-addition. n= 60 cells for CM4620 treated cells. **(B,C)** Analysis of SOCE in male and female microglia cultured separately from individual postnatal mice. Microglia isolated from postnatal pups were cultured separately (from each mouse) for 14 days. SOCE was initiated by depleting ER Ca^{2+} stores with TG as described above. 2 μM La^{3+} was added to the extracellular Ringer's solution at the indicated times. The right graph summarizes the rate of Ca^{2+}

influx following re-addition of extracellular Ca^{2+}. n=84 (males, no tamoxifen) and n=96 (males, tamoxifen). n=109 (females, no tamoxifen) and n=56 (females, tamoxifen) ***: p < 0.0001 by Mann-Whitney Rank sum test. **(D)** Example traces of Ca^{2+} responses in WT (Orai1$^{fl/fl}$) and Orai1 KO (Orai1$^{fl/fl\ CX3CR1\text{-}Cre/ERT2}$) microglia stimulated with LPS (1µg/ml). Ca^{2+} oscillations were summarized by counting the number oscillations that exceeded 20 nM. n=39 cells (Orai1$^{fl/fl}$) and n=21 cells (Orai1$^{fl/fl\ CX3CR1\text{-}Cre/ERT2}$). ***: p<0.001 by unpaired student's T-test with Welch Correction.

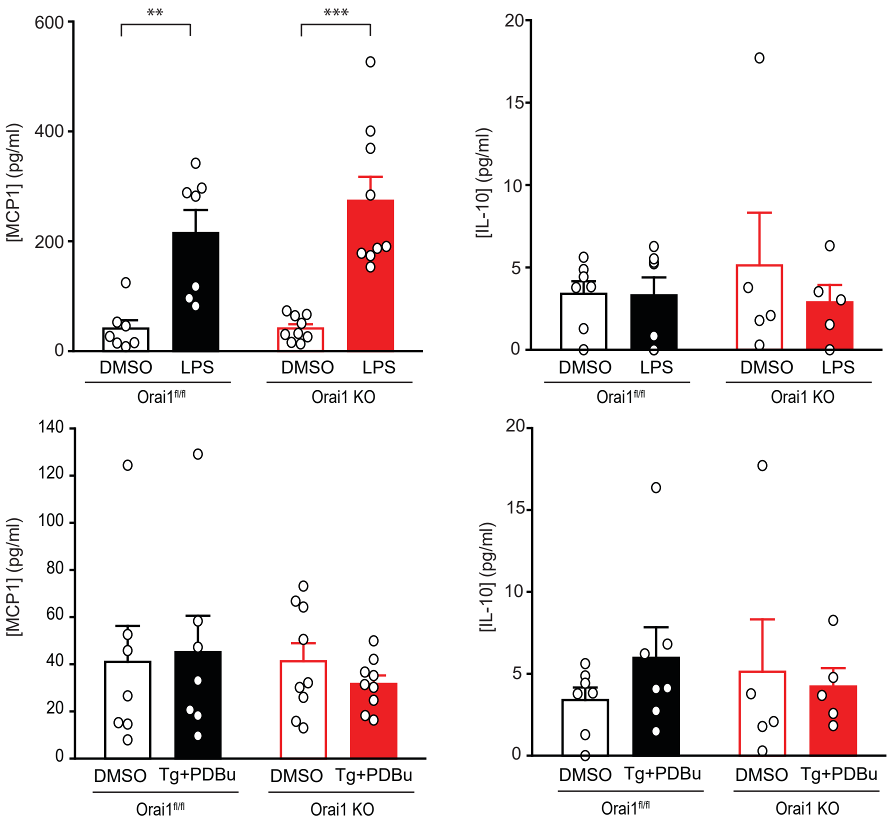

Figure. S3. MCP1 and IL-10 are not regulated by Orai1. MCP1 and IL-10 levels were assessed in the supernatant 18 hours following cell stimulation via ELISA. n=5-9 measurements from 10-18 mice. **: p<0.01 and ***: p<0.001 by two-way ANOVA followed by Tukey test for comparison between multiple groups. Deletion of Orai1 in microglia does not affect induction of MCP1. Moreover, IL-10 is not affected by genotype or treatment.

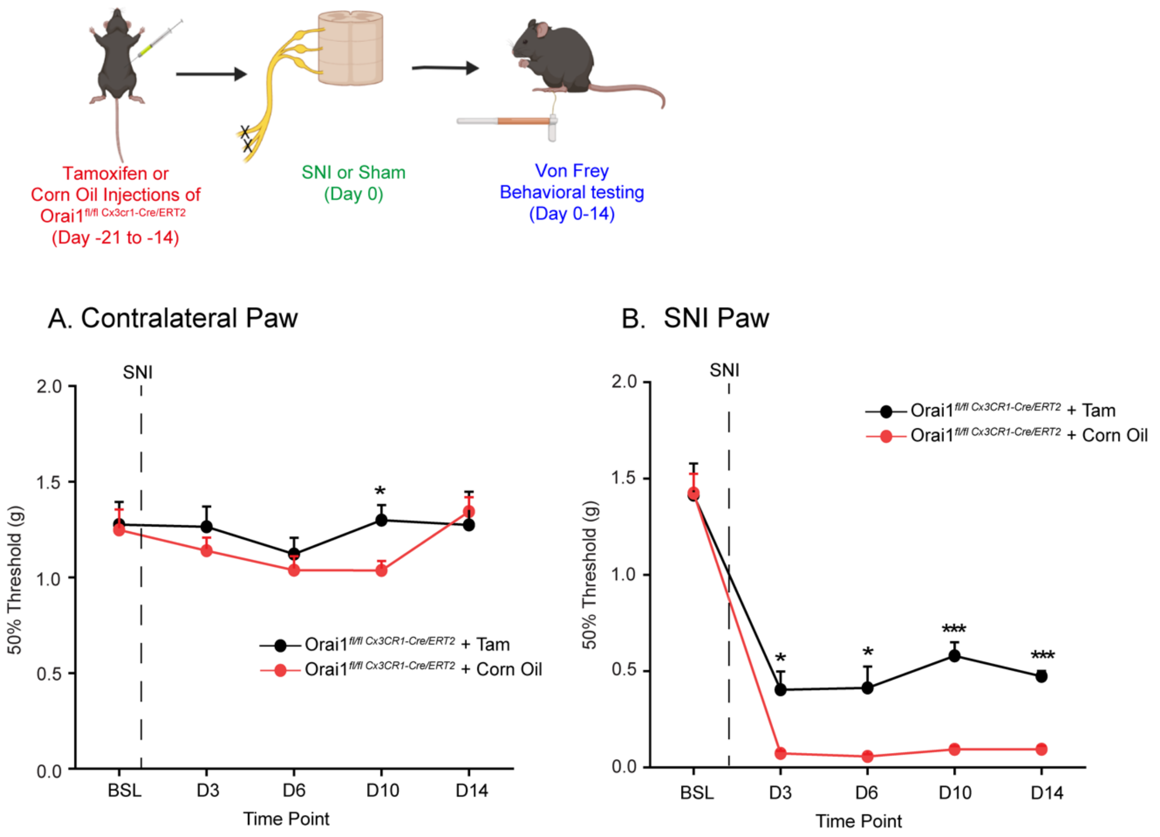

Figure S4. Analysis of allodynia in male Orai1$^{fl/fl\ CX3CR1\text{-}Cre/ERT2}$ mice with and without tamoxifen exposure. Mechanical sensitivity measured by von Frey thresholds in male Orai1$^{fl/fl\ CX3CR1\text{-}Cre/ERT2}$ mice injected with either tamoxifen or vehicle alone (corn oil). The dotted line indicates that day when SNI was performed. Only Orai1$^{fl/fl\ CX3CR1\text{-}Cre/ERT2}$ mice that received tamoxifen are protected from allodynia indicating that the pain protection is not due to Cre expression alone and that Orai1 deletion is required. n=9 (corn oil) and n=8 (tamoxifen). *: $p < 0.05$; ***:$p < 0.001$ by unpaired T-test comparing male Orai1$^{fl/fl\ CX3CR1\text{-}Cre/ERT2}$ mice with and without tamoxifen administration for each time point.

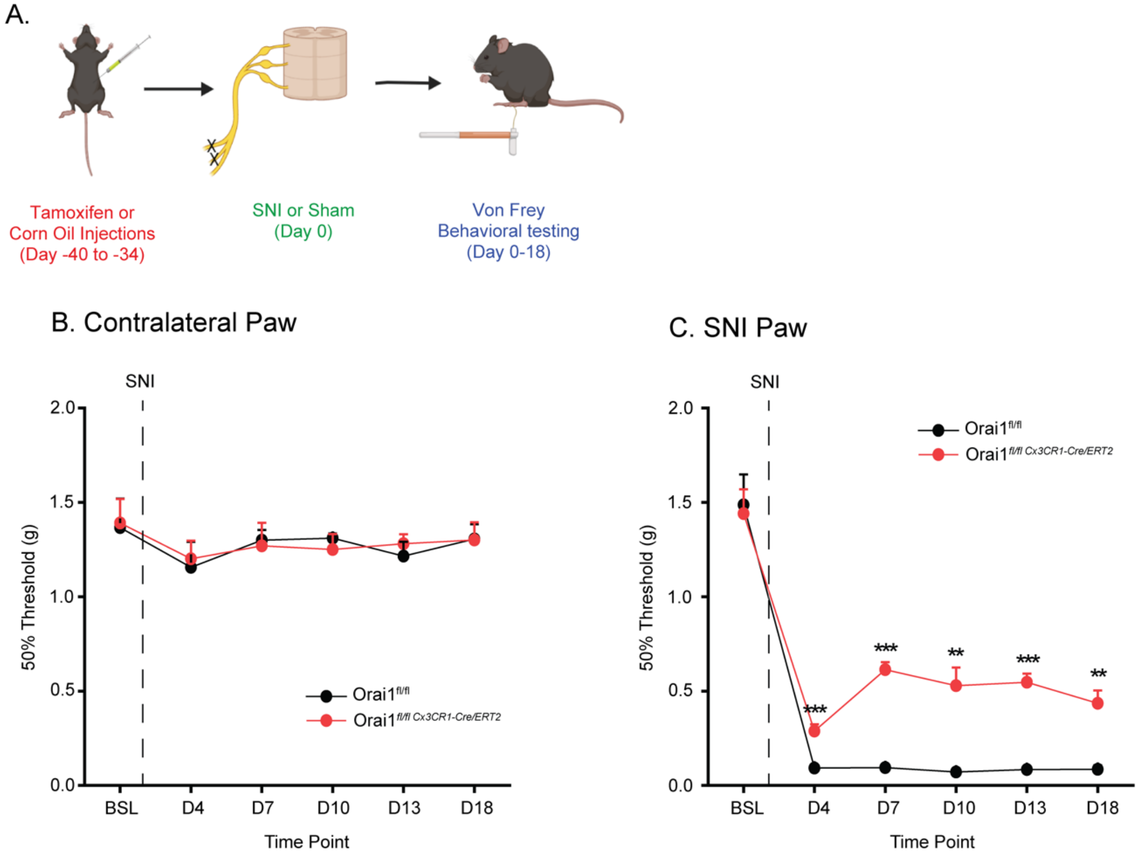

Figure S5. Analysis of pain hypersensitivity in male Orai1$^{fl/fl\ CX3CR1\text{-}Cre/ERT2}$ mice with longer waiting period after tamoxifen administration. Mechanical sensitivity measured by von Frey thresholds in male Orai1$^{fl/fl\ CX3CR1\text{-}Cre/ERT2}$ mice injected with either tamoxifen or vehicle (corn oil). The dotted line indicates that day when SNI was performed. Following the last tamoxifen injection, 34 days were allowed prior to SNI. Paw withdrawal thresholds were measured for 18 days following SNI. Orai1$^{fl/fl\ CX3CR1\text{-}Cre/ERT2}$ mice administered with tamoxifen are partially protected from allodynia throughout the measurement period even at 54 days after tamoxifen injection suggesting that the pain mitigation is not due to infiltration of peripheral monocytes expressing CX3CR1. n=6 (Orai1$^{fl/fl}$; males) and n=7 (Orai1 $^{fl/fl\ CX3CR1\text{-}Cre/ERT2}$; males). **: $p < 0.01$; ***: $p < 0.001$ by unpaired T-test comparing each time point.

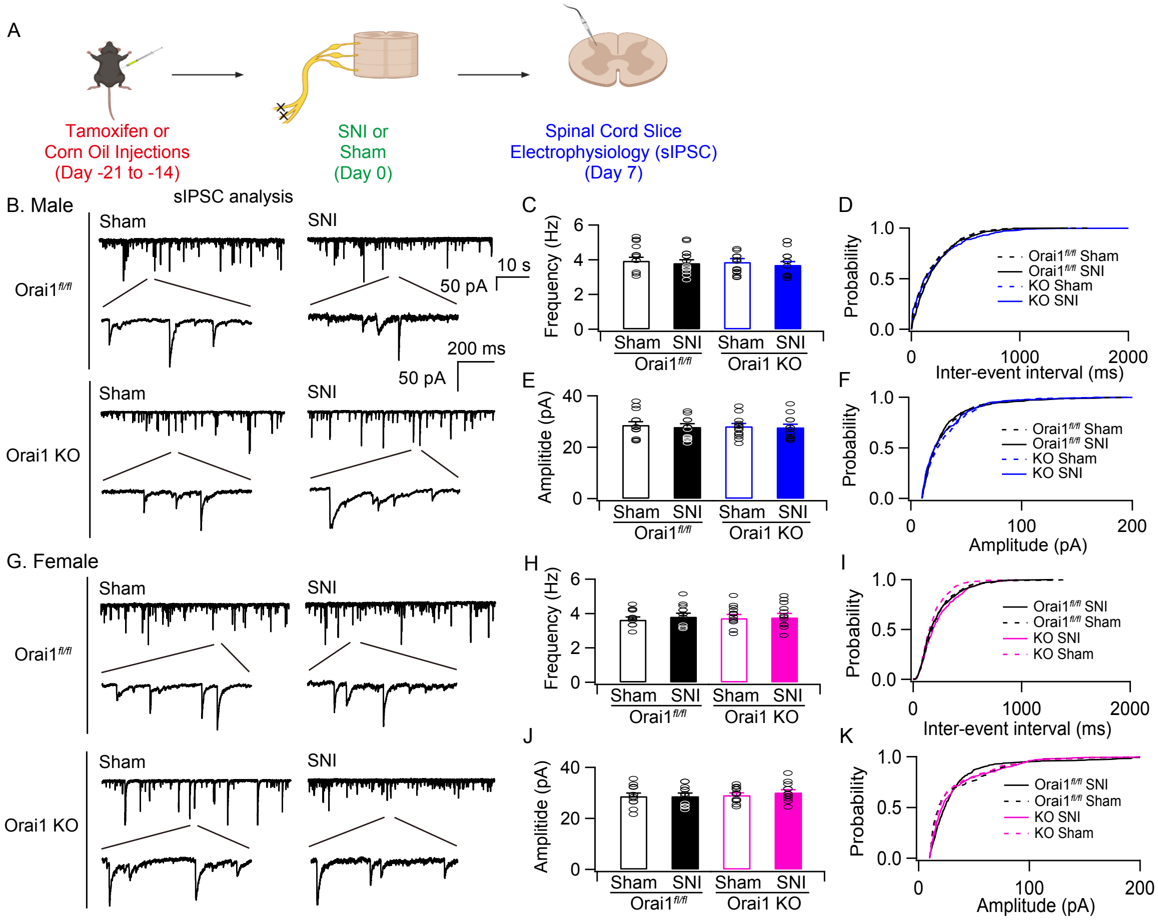

Figure S6. SNI does not affect the frequency or amplitude of spontaneous IPSCs. (A) Schematic of the experimental plan. **(B)** Examples of sIPSCs recorded from dorsal horn neurons of L4 spinal cord slices (at -70 mV) in lamina II. Excitatory synaptic transmission was blocked by the AMPA and NMDA receptor blockers, NBQX and D-APV, respectively. **(C)** Bar graphs (mean±sem) summarizing the sIPSC frequency. **(D)** Cumulative probability distribution of sIPSCs in the indicated groups. **(E)** Summary (mean±sem) of the amplitude of the sIPCS. **(F)** Cumulative probability distribution of the sIPSC amplitudes. **(G)** Example sIPCS traces from slices from female mice. **(H, J).** Summary of the frequency and amplitude of sIPSCs in female mice in the indicated groups. **(I,K)** Cumulative probability distributions of the sIPSC frequency and amplitudes. Cell counts are as follows: male WT mice: n=17 cells (sham), n=17 (SNI). Male Orai1 cKO mice: n=17 cells (sham), n=17 cells (SNI). Female WT mice: n=12 cells (sham), n=12 cells (SNI). Female Orai1 cKO mice: n=12 cells (sham), n=12 cells (SNI). Statistical analysis using two-way ANOVA followed by Tukey test for comparison of multiple groups.

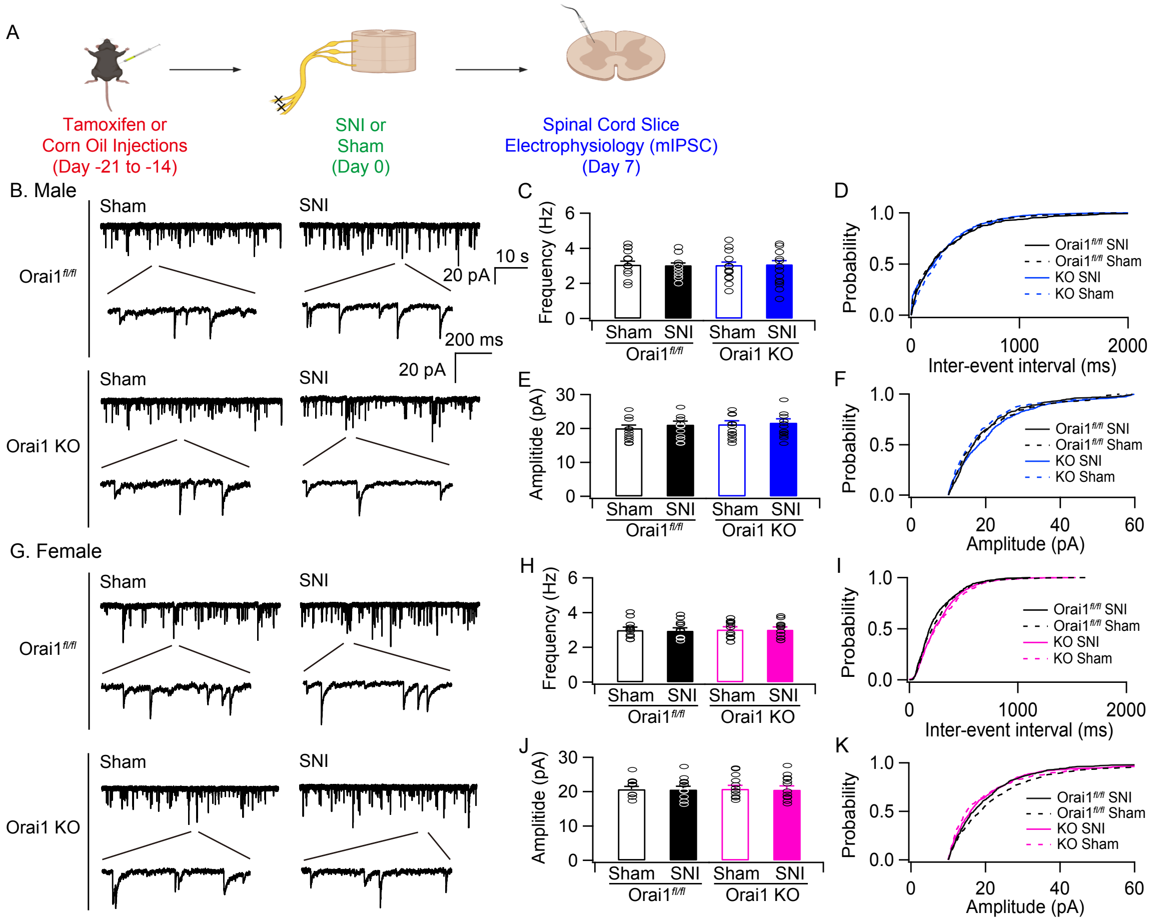

Figure S7. SNI does not affect the frequency or amplitude of miniature IPSCs. mIPSCs were recorded in the whole-cell patch-clamp configuration (at -70 mV) from a lamina II neuron in the dorsal horn of the L4 spinal cord in the presence of 1 μM TTX to block action potentials. Excitatory synaptic transmission was blocked by the AMPA and NMDA receptor blockers, NBQX and D-APV, respectively. **(B,G)** Example traces of mIPSCs at low and high time resolutions. **(C,E)** Bar graphs show the frequency and amplitude of the mIPCS in the indicated conditions. **(D,F)** Cumulative probability distributions of the inter-event intervals and amplitudes of the mIPSCs in WT and Orai1 cKO male mice. **(H,J)** Summary of the frequency and amplitude of the mIPCS in female mice in the indicated conditions. **(I,K)** Cumulative probability distributions of the inter-event intervals and amplitudes of the mIPSCs in WT and Orai1 cKO female mice. n=17 (male); n=12 (female). Statistical analysis was done using two-way ANOVA followed by Tukey test for multiple comparisons.

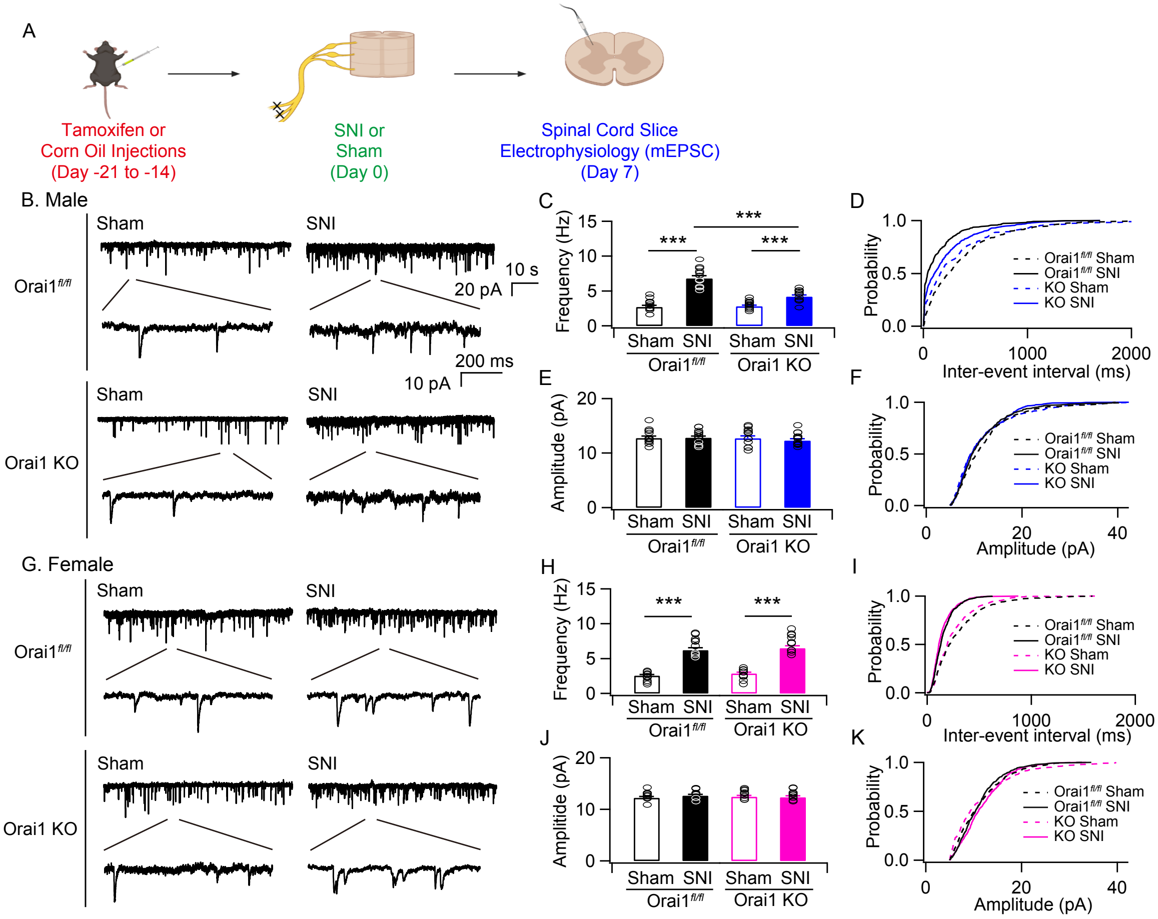

Figure S8. The SNI-induced increase in the frequency of miniature EPSCs is mitigated in male Orai1 cKO mice. (A) A schematic of the experiment. WT or Orai1 cKO mice (7-8 weeks old) were subjected to SNI or sham surgery. 7 days following SNI, animals were euthanized and the spinal cords isolated for slice electrophysiology. **(B,G)** mEPSCs recorded in the whole-cell patch-clamp configuration (-70 mV) from a lamina II neurons in the dorsal horn of the L4 spinal cord. TTX (1 µM) was perfused on the slices in all solutions to block action potentials. The traces show examples of mEPSCs at low and high time resolutions. **(C,E)** Bar graphs summarize the frequency and amplitude of mEPCS in the indicated conditions. SNI causes increases in the frequency of mEPSCs which is occluded in male mgOrai1 KO mice. There is no change in the amplitude of the events. **(D,F)** Cumulative probability distributions of the inter-event intervals and amplitudes of the mEPSCs in WT and mgOrai1 KO mice. The inter-event interval is shifted towards longer intervals consistent with increases in the frequency of mEPSCs in mice subjected to SNI. **(H,J)** Summary of the change in frequency and amplitude of mEPSCs in female mice. Here, the frequency of mEPSC is unaffected and comparable to SNI-administered WT mice. **(I-K)** Cumulative probability distributions of the inter-event intervals and amplitudes of mEPSCs in WT and female cKO mice. n=13 (male), n=10 (female). ***: p<0.001 by ANOVA followed by Tukey test for comparison between multiple groups.

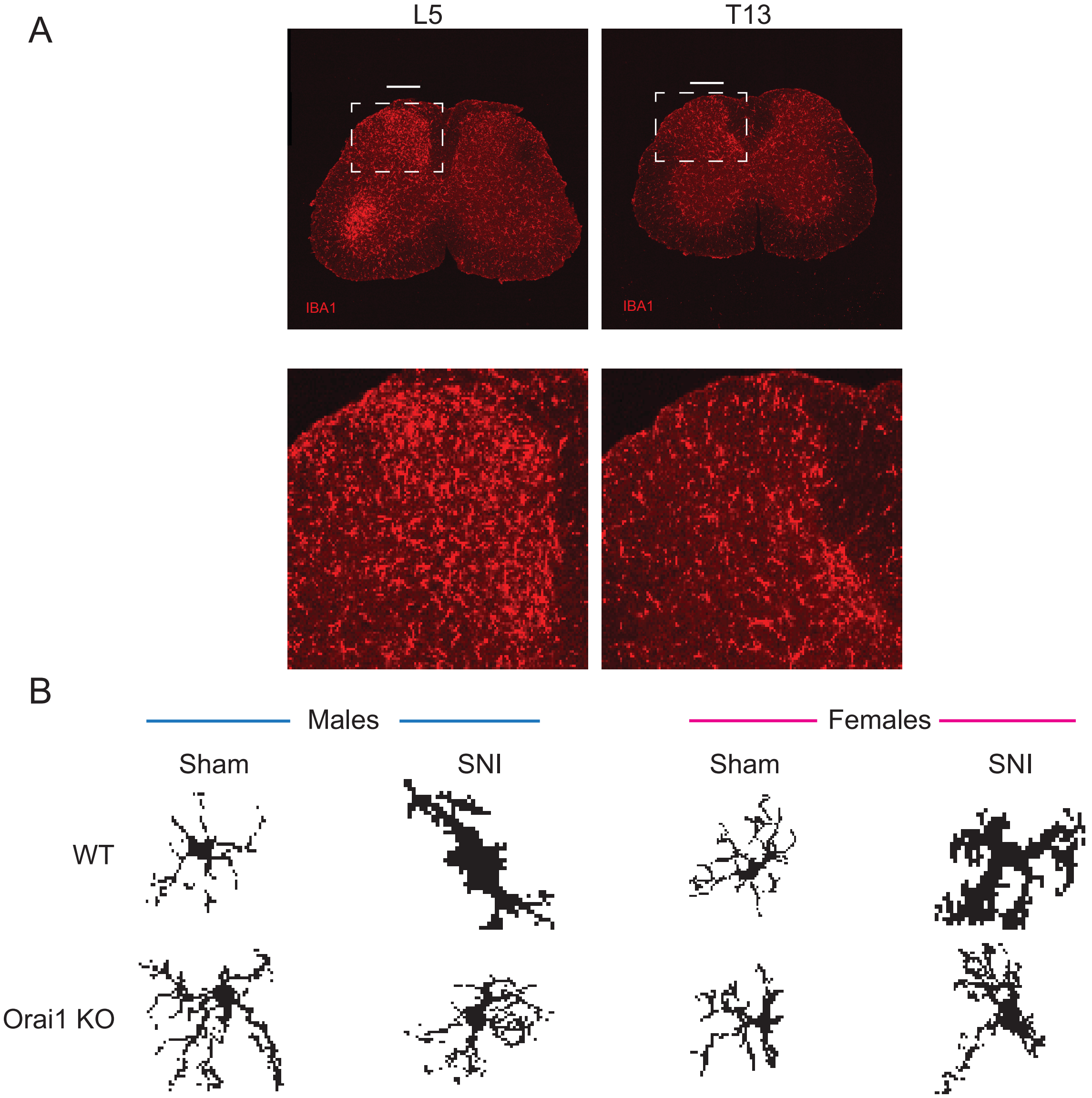

Figure S9. IBA1 staining in the lumbar and thoracic spinal Cord after SNI. (A) Images of the L5 and T13 spinal cord sections from a WT mouse after SNI. Scale bar: 250 µm. The lower row of images depict the magnified boxes in the upper row. IBA1 labelling increases in the dorsal and ventral horns of the on the ipsilateral side of injury in the L5 but not in the T13 sections. **(B)** Binary mask images of isolated microglia from sections stained for IBA1 expression in Figure 6 highlight the morphology of microglia in the indicated conditions. Microglial from sham and Orai1 cKO mice have a more ramified appearance with long processes compared to WT SNI-treated mice.

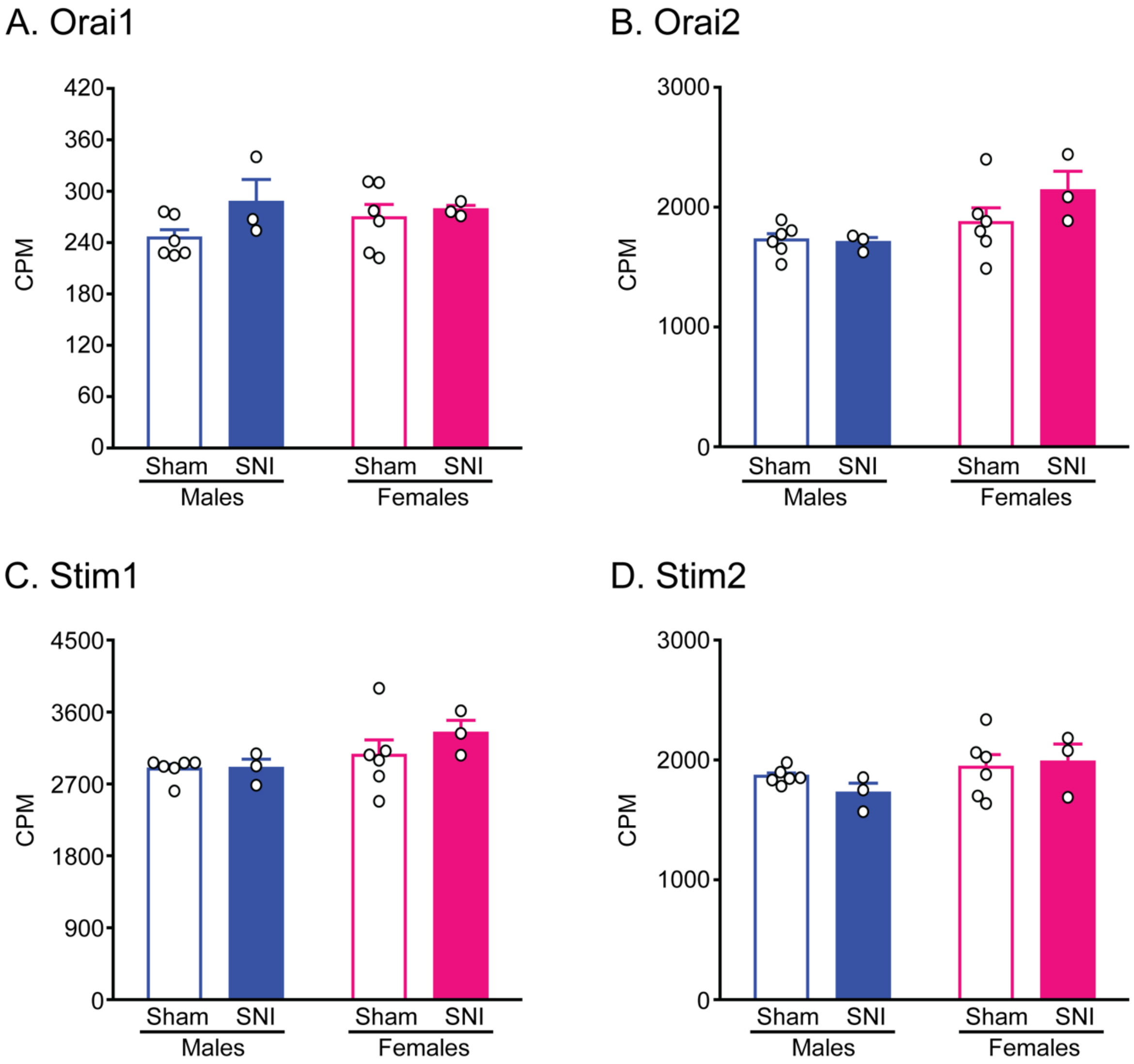

Figure S10. Expression of SOCE genes is unchanged after SNI. (A-D) Bar graphs of the normalized counts-per-million (CPM) reads from each sample (L4-L5 spinal cord from one mouse). The SNI data was obtained from the ipsilateral dorsal horn of WT mice subjected to SNI. The samples in the sham group contain both ipsilateral and contralateral dorsal horn expression analysis. There is no significant difference in any of the genes between sham and SNI groups. Additionally, no difference was seen between male and female WT mice after SNI. *(A)* Orai1, *(B)* Orai2, (*C)* STIM1, *(D)* STIM2. (two-way ANOVA analysis of CPM).

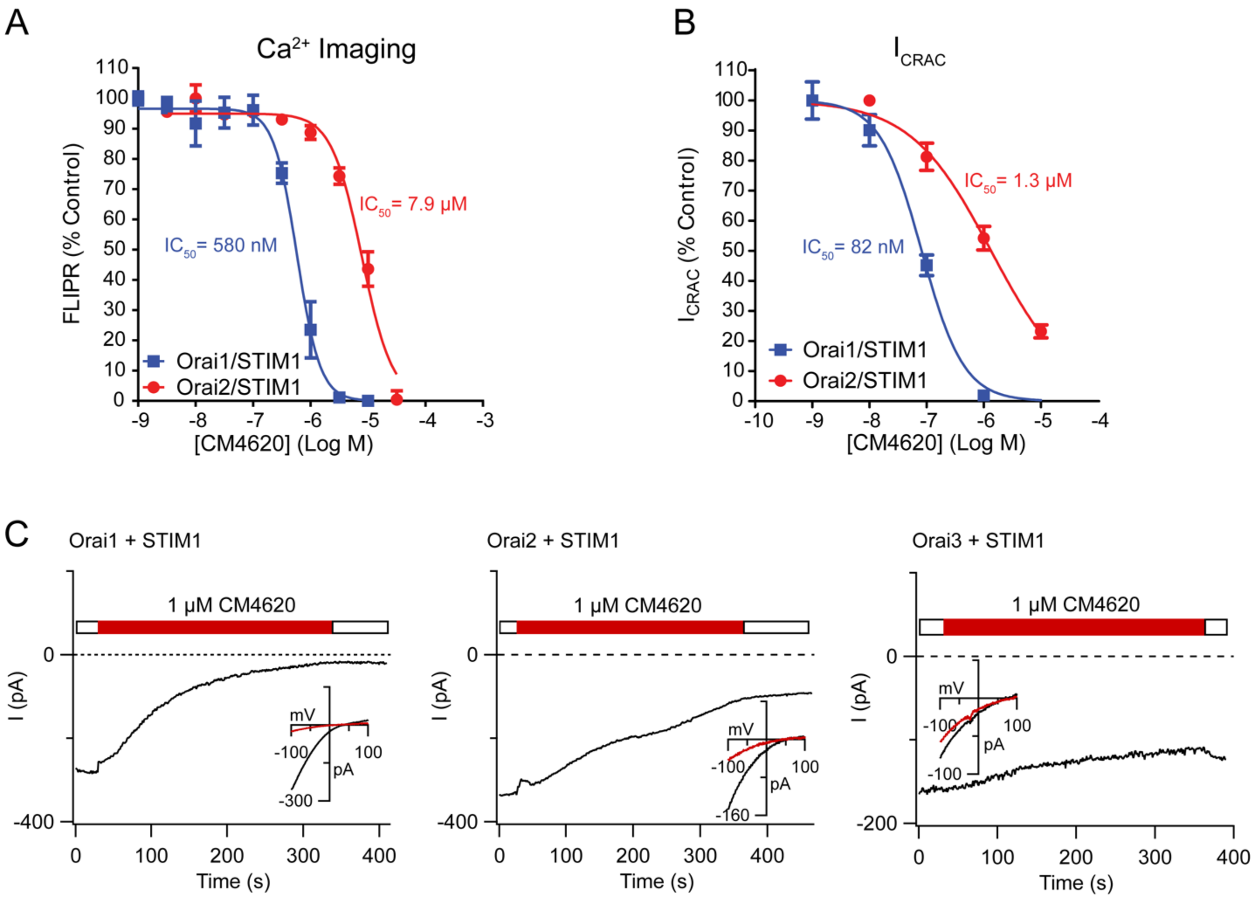

Figure S11. Dose dependence of CM4620 inhibition of SOCE and Orai1 specificity. (A, B) Dose-response of CM4620 inhibition of SOCE and CRAC currents measured in HEK293 cells expressing either Orai1 or Orai2 together with STIM1. **(C).** Example CRAC current traces showing inhibition of I_{CRAC} by 1 μM CM4620. Patch-clamp recordings were carried out in HEK293 cells expressing either Orai1, Orai2, or Orai3 with mCherry-STIM1. The insets show the current-voltage relationship of the indicated CRAC current before and after CM4620 administration.

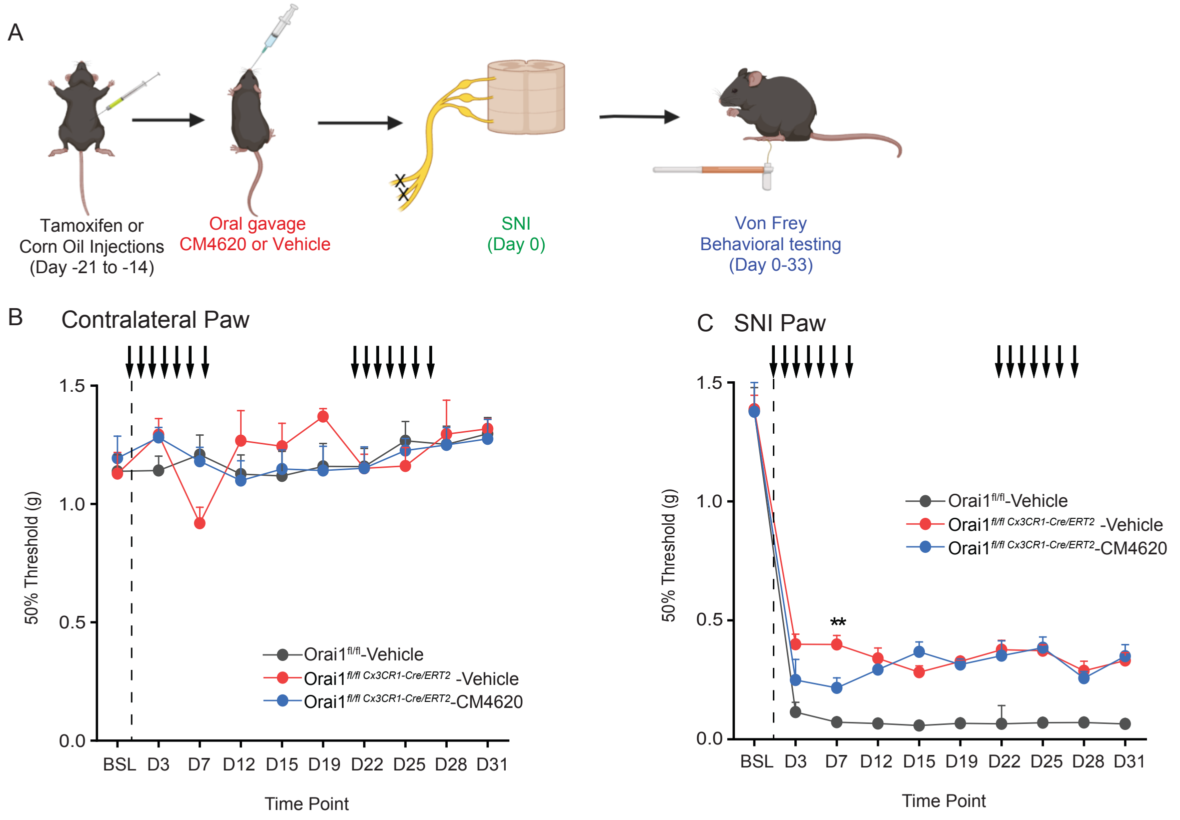

Figure S12. Pain hypersensitivity in male Orai1$^{fl/fl\ CX3CR1\text{-}Cre/ERT2}$ mice treated with CM4620. Mechanical sensitivity was measured by von Frey thresholds in Orai1$^{fl/fl\ CX3CR1\text{-}Cre/ERT2}$ and Orai1$^{fl/fl}$ mice injected with tamoxifen. The dotted line indicates the day when SNI was performed. CM4620 (10 mg/kg) was delivered by oral gavage for 7 days as indicated by the arrows. von Frey thresholds were determined at periodic intervals for 31 days after SNI. CM4620 was stopped after day 8 for 14 days and resumed on day 22. n=7 mice, Orai1$^{fl/fl}$ (3 males, 4 females); n=6 mice Orai1$^{fl/flCX3CR1\text{-}Cre}$ (all male); n=6 mice, Orai1$^{fl/flCX3CR1\text{-}Cre}$ + CM4620 (all male). **: $p < 0.01$ by two-way ANOVA followed by Tukey test for comparison between multiple groups at each time point.
